# An Analysis of Prescribed Fire Activities and Emissions in the Southeastern United States from 2013 to 2020

**DOI:** 10.3390/rs15112725

**Published:** 2023-05-24

**Authors:** Zongrun Li, Kamal J. Maji, Yongtao Hu, Ambarish Vaidyanathan, Susan M. O’Neill, M. Talat Odman, Armistead G. Russell

**Affiliations:** 1 School of Civil and Environmental Engineering, Georgia Institute of Technology, Atlanta, GA 30332, USA;; 2 National Center for Environmental Health, Centers for Disease Control and Prevention, Atlanta, GA 30341, USA;; 3 United States Department of Agriculture Forest Service, Pacific Northwest Research Station, Seattle, WA 98103, USA;

**Keywords:** biomass burning, wildland fire, prescribed burning emission, BlueSky, FINN

## Abstract

Prescribed burning is a major source of a fine particular matter, especially in the southeastern United States, and quantifying emissions from burning operations accurately is an integral part of ascertaining air quality impacts. For instance, a critical factor in calculating fire emissions is identifying fire activity information (e.g., location, date/time, fire type, and area burned) and prior estimations of prescribed fire activity used for calculating emissions have either used burn permit records or satellite-based remote sensing products. While burn permit records kept by state agencies are a reliable source, they are not always available or readily accessible. Satellite-based remote sensing products are currently used to fill the data gaps, especially in regional studies; however, they cannot differentiate prescribed burns from the other types of fires. In this study, we developed novel algorithms to distinguish prescribed burns from wildfires and agricultural burns in a satellite-derived product, Fire INventory from NCAR (FINN). We matched and compared the burned areas from permit records and FINN at various spatial scales: individual fire level, 4 km grid level, and state level. The methods developed in this study are readily usable for differentiating burn type, matching and comparing the burned area between two datasets at various resolutions, and estimating prescribed burn emissions. The results showed that burned areas from permits and FINN have a weak correlation at the individual fire level, while the correlation is much higher for the 4 km grid and state levels. Since matching at the 4 km grid level showed a relatively higher correlation and chemical transport models typically use grid-based emissions, we used the linear regression relationship between FINN and permit burned areas at the grid level to adjust FINN burned areas. This adjustment resulted in a reduction in FINN-burned areas by 34%. The adjusted burned area was then used as input to the BlueSky Smoke Modeling Framework to provide long-term, three-dimensional prescribed burning emissions for the southeastern United States. In this study, we also compared emissions from different methods (FINN or BlueSky) and different data sources (adjusted FINN or permits) to evaluate uncertainties of our emission estimation. The comparison results showed the impacts of the burned area, method, and data source on prescribed burning emission estimations.

## 1. Introduction

The burned area from wildland fires in the United States has increased in recent decades [1], with the frequency and severity of wildland fires continuing to grow under a changing climate [2–4]. Moreover, the greenhouse gases emitted from fires have positive feedback on global warming, leading to more pronounced and persistent climate-related impacts [5]. Prescribed burning, which is a type of planned burning operation and falls under the broader definition of wildland fires, is introduced to recreate the natural fire regimes for a healthy ecosystem and mitigate the risk of severe wildfires by reducing hazardous fuels. Given that burns are planned and conducted by experts and emissions are typically lower compared to wildfires [6], prescribed burning impacts may be less hazardous. However, the effects of prescribed burning are particularly felt in the southeastern United States, where prescribed burning has been traditionally used for land management in both private and public lands [7,8].

Emissions from fires are typically estimated using burned area, fuel bed information (e.g., type and amount of fuels), the efficiency of combustion, and emission factors [9]. Meteorological conditions such as moisture can also affect fire emissions [10–12]. The information used to estimate fire emissions can be obtained from either ground-based fire datasets or remote sensing techniques. Since ground-based fire datasets are not provided in all locations, remote sensing techniques are typically used to provide fire information for global or regional fire emission products. Based on remote sensing technology, several satellite-derived products are employed to estimate emissions from wildland fires. The Blended Polar Geo Biomass Burning Emissions Product (Blended-BBEP) [13], Global Fire Emissions Database (GFED4s) [14], and Fire INventory from NCAR (FINN) [15,16] use fire radiative power (FRP), which reflects the rate of thermal energy released from fires, to estimate burned area. The fire emissions are then derived from the estimated burned area. Products such as the Blended Global Biomass Burning Emissions Product version 3 (GBBEPx v3) [17] directly estimate the emission from fire radiative energy (FRE), which measures the total amount of energy released during biomass combustion episodes. The FRE is expected to have a linear relationship with fuel consumption [18]. Satellite-derived products provide historical and real-time global fire emission estimates, though cloud cover or resolution of the satellite imagery leads to uncertainty. Prescribed burning, which is typically smaller in size and is designed to burn at a low intensity and/or as an understory burn, is often missed by satellites [7]. Apart from satellite-derived products, burn permit records provide prescribed fire information. Prescribed burning permits report the burned area, location, and timing of the prescribed burning, and this information can be used for emission estimation. Since the burned areas of fires are crucial in emission estimation for permits or some satellite-derived products, comparisons between the burned area from prescribed burning permits and satellite-derived products can inform the degree of uncertainty of both methods. Koplitz et al. [19] compared the annual total burned area from the National Interagency Fire Center (NIFC), which is a ground-based record, with different satellite products in different regions of the United States. The results indicate that burned areas from different products in the northeastern and northern United States have a high correlation, while the southeastern and southern United States have discrepancies due to the uncertainty in the burned area from small fires. Zeng et al. [20] compared monthly burned area from a bottom-up database VISTA with Terra MODIS at the state level, reporting correlation coefficients (R^2^) of 0.57 and 0.52, respectively, for all fire types and prescribed burning, the latter of which is not separated from other fire types in MODIS. Huang et al. [21] compared the actual burned area, which was obtained by phone call surveys of land managers and prescribed burn contractors, with burned areas from permits in Georgia, and found a high correlation (R^2^ > 0.64) between the two. The study also compared burned areas from permits, Blended-BBEP, and GFED4s in Georgia and Florida at a relatively coarse resolution (state-based or county-based). The correlation between satellite-derived and permit-reported burned areas is relatively low compared to the correlation between surveyed land and permits provided to the burned area. To enhance the accuracy of emissions estimation and burned area comparisons, it is important to differentiate between burn types. However, this is often not possible with satellite data as fires are typically detected by thermal energy, which makes it difficult to distinguish between prescribed burns and wildfires. Despite this, some recent studies and emission products have made progress in differentiating burn types. For instance, CFIRE [22] used a combination of remote sensing data and permit records to provide burn-type information, allowing for separate calculations of emissions based on different types of wildland fires. McClure [23] employed a spatiotemporal clustering algorithm to estimate the growth pattern and lasting duration of wildfires, suggesting that it is possible to separate long-term fires from prescribed burns. In this work, we differentiate prescribed burns from wildland fires in FINN and compare the daily burned areas of prescribed burns from FINN to permits under different spatial resolutions.

The ultimate goal of the research is to generate three-dimensional prescribed burning emissions for chemical transport modeling. The study includes three parts. In the first part, we developed a method to identify fire types by differentiating agricultural burning, wildfires, and prescribed burning in FINN data. In the second part, we compared the burned area for prescribed burning from FINN and permits by developing and applying novel algorithms to match records from these two sources. A linear regression model was employed to capture the relationship between the burned area from FINN and permits and adjust the burned area of prescribed burning from FINN. In the third part, we estimated the magnitude and vertical structure of emissions based on the adjusted burned area and BlueSky Smoke Modeling Framework [24]. We compared the emissions magnitudes from different methods (FINN and BlueSky) and from different data sources (adjusted FINN and permits) to evaluate and understand the uncertainties in prescribed fire emissions. Then, we used a plume rise model incorporated in BlueSky to generate vertical emission profiles, which we combined with emission magnitudes to obtain three-dimensional prescribed burning emissions. Our results and the following discussion highlight the various challenges associated with obtaining fire activity information suitable for estimating prescribed burning emissions.

## 2. Materials and Methods

### 2.1. State-Prescribed Burning Permit Records

Prescribed burning permits, which contain some of the fire activity information needed for fire emission estimates, were obtained from forestry agencies in Florida [25], South Carolina [26], and Georgia [27]. Georgia permit data covered 2015–2020. Florida and South Carolina permit data covered 2013–2020. Permit records in Florida and South Carolina provided detailed information on prescribed burns including the latitude and longitude of prescribed burn locations, burned area, and start time of prescribed burning, which were used in the following analysis. The data had missing values and wrong locations due to human error, so we removed the permits for which burned area was invalid (zero or missing), or the location was out of the state boundary (less than 1.0%). For Georgia, the latitude and longitude of prescribed burning were provided for a small portion of the permits. Most locations of burns were provided by address. We conducted address geocoding using Google Maps [28] for the permits that did not have latitude or longitude. A total of 4.6% of the addresses of the permits could not be matched via geocoding since the descriptions were ambiguous or erroneous; therefore, they were removed.

### 2.2. Fire INventory from NCAR (FINN)

Satellite-derived data is from FINN version 2.5 [15,16] in our study. FINN version 2.5 employed a spatial clustering algorithm to merge detected active fires since different active fires from satellites can correspond to a single fire event. Additionally, the clustering algorithm is utilized to combine fire detections from VIIRS and MODIS. FINN utilizes the combined outputs to estimate the burned area [16]. Since VIIRS has a higher resolution (375 m) than MODIS (1 km), FINN version 2.5 can detect fires with smaller burned areas [29]. This is particularly important for estimating emissions from prescribed burns, which are typical for low intensity and/or occur as understory burns. In this study, we extract all wildland fires from FINN version 2.5 that were detected in the southeastern United States (as defined in Figure S25) from 2013 to 2020. The burned area of prescribed fires is separated by a burn-type differentiation algorithm and compared with the burned area reported by the permits.

### 2.3. BlueSky Smoke Modeling Framework

The BlueSky Smoke Modeling Framework [24] provides multiple modeling options to estimate fuel type, fuel load, fuel moisture, fuel consumption, emissions, and smoke height. In this study, we employ BlueSky to estimate emissions and generate three-dimensional emission data for chemical transport modeling. North American Mesoscale Forecast System (NAM) 12 km [30] data is used to provide meteorological conditions for running BlueSky. The Weather Research and Forecasting Model (WRF) [31], with a 12 km resolution, provides meteorological conditions for the dates when NAM is missing. For BlueSky simulations, the 1 km Fuel Characteristic Classification System (FCCS) [32] provides detailed descriptions of the fuel beds; the National Fire Danger Rating System (NFDRS) [33] estimates fuel moisture (which affects fuel consumption); the CONSUME model [34] and Prichard–O’Neill’s emission factors [35] are used to calculate consumption and emissions; and the Fire Emission Production Simulator (FEPS) with Briggs plume top behaviors [36] estimates the vertical structure of emissions. Neither prescribed burn permits nor FINN provides a complete start hour or end hour of fires, so we assume that prescribed burning starts at 11 am local time and ends before 6 pm local time since prescribed burning is typically executed during the daytime, and there is a lag between when a fire crew starts work and the burn begins. The duration of prescribed burning is estimated based on the burned area (Table S1).

## 3. Methods

### 3.1. Burn-Type Differentiation

#### 3.1.1. Agricultural Burning Identification

Agricultural burning includes planting preparation burning, crop residue burning, and stubble burning. Fuel load and emission factors vary for different crop residues [37]. Agricultural burning has different emission patterns than prescribed burning due to the differences in fuel type, fuel load, timing, and frequency, so it is important to differentiate agricultural burns from prescribed burns. Georgia does not provide detailed burning purposes in its permit records, and Florida does not have burn type data for 2017 and 2020. Satellite-derived FINN data does not differentiate burn types. To identify agricultural burns, we utilize the National Land Cover Database (NLCD), which has a 30 m resolution and is updated every 2 to 3 years [38]. We use 2013, 2016, and 2019 NLCD data to provide the land cover type of fires for their following years (e.g., 2013 for 2013–2015). For each fire, we assume a square shape with the same area as the burned area. The dominant land cover type in the square is assumed to be the land cover type for the fire. For fires that happened in areas classified as agriculture, we use agricultural burn estimation. Fires in open water or barren land in FINN or permits are removed as they are likely the wrong coordinates in FINN or permit records.

#### 3.1.2. Wildfire Detection Algorithm

FINN data includes all fires detected by MODIS and VIIRS satellites, both for wildfires and prescribed, while permits are only for prescribed burns. Therefore, matching FINN data with permit data requires the removal of wildfires from FINN. In this research, we focus on detecting larger wildfires. Prescribed burns typically start and end on the same day, while wildfires can last multiple days, so we assume the fires that have more than a one-day duration are wildfires. The duration of the fires can be calculated by temporally tracking each fire in FINN. In other words, if FINN detects fires in the same region (the distance between detected fires in the region is less than the selected clustering distance) for consecutive days, the algorithm will mark all fires in that region during the period as wildfires. Since wildfires can also spread over long distances in one day, FINN may identify a wildfire as several fires. This segregation problem could lead to underestimations of the size of wildfires if we just track the wildfires temporally, so a spatial clustering algorithm is applied to cluster FINN fire records that are close to each other on the same day (Algorithm S1). In this spatial and temporal clustering algorithm, we use 1000 m as the spatial clustering distance and 800 m as the temporal clustering distance for clustering the different FINN records into a wildfire based using the elbow method [39]. The elbow method is widely used to determine the optimal number of clusters in a clustering algorithm, and the elbow point of the clustering parameters (spatial and/or temporal clustering distance) versus the number of clusters plot represents a suitable balance between clustering accuracy and model complexity. As a result, we have selected the elbow point as the optimal parameter setting for our clustering algorithm. It is recognized that wildfires can happen under similar meteorological conditions as prescribed burns, and some wildfires are small and extinguished in a short time, leading to potential misclassification.

### 3.2. Matching FINN-Prescribed Burning Records with Permits

FINN is a satellite-derived product and estimates the burned area with the help of fire radiative power detected from MODIS and VIIRS. The approach has several limitations due to a lack of information on combustion completeness or fuel loading [40]. Additionally, cloud cover and thick smoke due to large fires can affect radiative power detection by satellites [41]. Permit records are reported when land managers plan to execute prescribed burns and may not reflect the actual day a burn was accomplished. The burned area in permit records is estimated by land managers. To evaluate the disparity between FINN and permit estimates, we first removed agricultural burns from both datasets using an agricultural identification algorithm. Then, we applied a wildfire detection algorithm to remove any wildfires detected by FINN. Finally, we compared the prescribed burning burned area in permits and FINN using three types of matching: statewide, fire-to-fire, and grid-based.

#### 3.2.1. Statewide Matching

For statewide matching, we calculate the daily total burned area in each state as reported in the permits and calculated by FINN. The statewide daily total burned area reflects the temporal pattern of prescribed burns in selected states. The spatial distribution is not compared.

#### 3.2.2. Fire-to-Fire Matching

Fire-to-fire matching has the highest spatial resolution and is essential for event-based air quality modeling. We first match the FINN and permit records which have the nearest distance to each other (Algorithm S2). The matching algorithm is based on distance alone, leading to potential problems since the burn date of the permits can be different from the actual burn date. It is also possible that the closest pair of FINN and permit records may not be a match, as there is uncertainty in FINN and permit record location. Therefore, we implement an algorithm that can relax the date or distance requirements (Algorithm S3). If the difference in start date and distance between permits and FINN is less than specified values, the burns of FINN and the permit are considered as candidates for being matched pairs. Differences in burned area are included as an additional metric to select the best pair among the candidate-matched pairs.

#### 3.2.3. Grid-Based Burned Area Matching

As a third method, we generated the grid-based burned area by aggregating all the burns in a grid cell and found the relationship between FINN and permits. The method can partly solve the segmentation issues in FINN or the location uncertainties in FINN or permits. Meanwhile, the chemical transport model computes air quality at a grid-based resolution, so utilizing grid-based burned areas to generate grid-based emissions would not have significant impacts on the results of the air quality model. Here, we use a 4 km grid definition to generate grid-based, burned area fields. We conduct a sampling method that uses the averaged value of 3 by 3 grids to represent the center value of the 3 by 3 grids to address the potential for fires to occur near the grid boundaries.

## 4. Results

### 4.1. Burn-Type Differentiation

#### 4.1.1. Agricultural Burning Identification

We used Florida’s burn type data to evaluate the performance of the agricultural burning identification algorithm. There were 95,364 records in Florida permits labeled as agricultural burns, 65.35% of which were also identified as agricultural burns by the algorithm. On the other hand, 70,157 permit records were labeled as agricultural burns by the algorithm, of which 88.83% were validated by the burn type in permit records. The results showed that there was good agreement between information obtained from permits and the detection offered by the algorithm. Meanwhile, the algorithm underestimated the number of agricultural burns in Florida.

#### 4.1.2. Wildfire Detection

The wildfire algorithm needs two parameters: a spatial clustering distance and a temporal clustering distance for aggregating FINN fires. We tuned these two parameters separately and analyzed the spatial and temporal relationships in FINN data (Figure S1). For spatial clustering, we clustered the fires observed on the same day. The clustering started at around 250 m, which means FINN did not have any fires within less than 250 m of each other. The number of clusters was increasing, and the rate of increase slowed down after 1200 m. This result showed that most FINN records clustered in the 250 m to 1200 m range. For temporal clustering, we clustered the fires which occurred closer than a specific distance within a number of consecutive days, the duration of which was determined by the algorithm itself. The “elbow” (the maximum curvature, also known as the knee, for the temporal clustering curve in Figure S1) was around 800 m, which indicates that the number of clusters increased intensively when the distance threshold was less than 800 m. For wildfire detection, we clustered FINN fire records in space and time simultaneously by using our wildfire detection algorithm. Clustering parameters were tuned at the same time, and the elbow method [39] suggested 1000 m as the spatial clustering distance and 800 m as the temporal clustering distance (Figure S2). For wildfire detection algorithm evaluation, we used the number of matched wildfires between algorithm-detected wildfires and wildland fire location full history (WFIGS [42]) records in the United States from 2014 to 2020. As a matching method, we relaxed the distance (1500 m) and date (3 days) (Algorithm S3) by considering the uncertainty of the wildfire’s reported location and discovery date. A total of 3430 WFIGS records, whose burned area was higher than the minimum burned area of algorithm-detected wildfires, were selected. The algorithm detected 22,140 wildfires based on clustering FINN fires temporally and spatially, and 665 wildfires were identified by both WFIGS and our algorithm.

#### 4.1.3. Burn Types in FINN and Permits

From the burn-type differentiation algorithm, we differentiated agricultural burns for FINN and permits, and separated wildfires and prescribed burns for FINN. We estimated the number of prescribed burns and burned areas for each southeastern state based on FINN or permits (Figures S3, 1 and 2). In the southeastern United States, the primary type of burn was prescribed burn, except in Arkansas where agricultural burns were most prevalent. West Virginia and Virginia had the largest portion of wildfires among the southeastern states. Half of Florida’s permit records were associated with agricultural burning; however, prescribed burns accounted for a larger proportion of the burned area (79.40%). The percentages of the burned area from prescribed burning in Georgia and South Carolina were 79.00% and 88.70%, respectively.

### 4.2. Matching Prescribed Burning Records in FINN with Permits

**Figure 3.**
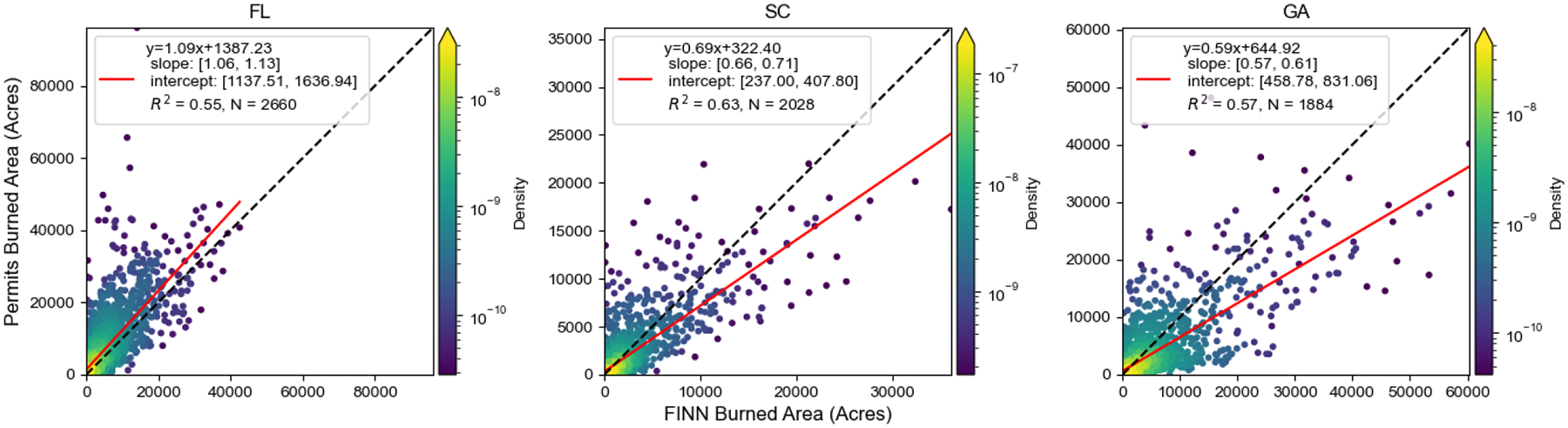
Statewide matching between FINN and permits in Florida, South Carolina, and Georgia. A linear regression with an intercept was conducted to fit the FINN burned area and permits burned area. The numbers of matching days were indicated as N values. Uncertainty of linear regression parameters was reported with a 95% confidence interval. Florida and South Carolina permits cover 2013–2020. Georgia permits cover 2015–2020. (State abbreviations: GA: Georgia; FL: Florida; SC: South Carolina).

We extracted prescribed burns from FINN and permits by the burn-type differentiation algorithm. To understand the relationship between the burned area from FINN and permits, we matched records at different spatial scales. Statewide matching showed a relatively strong correlation (R^2^ > 0.55) (Figures 3 and S4). Of note, the slope was higher than 1 in Florida and was lower than 1 for South Carolina and Georgia for both linear regression models, indicating that FINN underestimated the prescribed burned area in Florida and overestimated it in Georgia and South Carolina. The positive intercept in the linear regression model showed that FINN underestimated burned area when the daily burned area was small. Additionally, the temporal pattern of prescribed burns from FINN was consistent with permits (Figure S5). The peaks of the burned area were around February to May for these three states.

Although the regression model performed well when we matched FINN and permits in a coarse spatial scale, FINN and permits had poor correlation when we conducted a fire-to-fire matching with or without distance and date relaxation (Algorithms S2 and S3). For the nearest distance matching algorithm, we changed the selected distance threshold and evaluated the matching performance by R^2^ (Figure S6). The R^2^ was less than 0.05 even when we only considered a distance of less than 500 m between the FINN record and permit as a matched pair. The poor performance was partly due to uncertainty of location and start date for the permits, especially in Georgia, where coordinates of burns were estimated from ambiguous descriptions of addresses. Additionally, using distance as the single metric for matching had high uncertainty when there was more than one fire in FINN close to a permit record, and vice versa. Hence, we allowed some relaxations on the start date and included differences in the burned area as another metric for matching (Algorithm S3). By tuning the distance and start date, the matching performance was better than the previous method but was still poor (Figure S7). The disparity of performance between statewide matching and fire-to-fire matching showed the matching performance was sensitive to spatial resolution.

**Figure 4.**
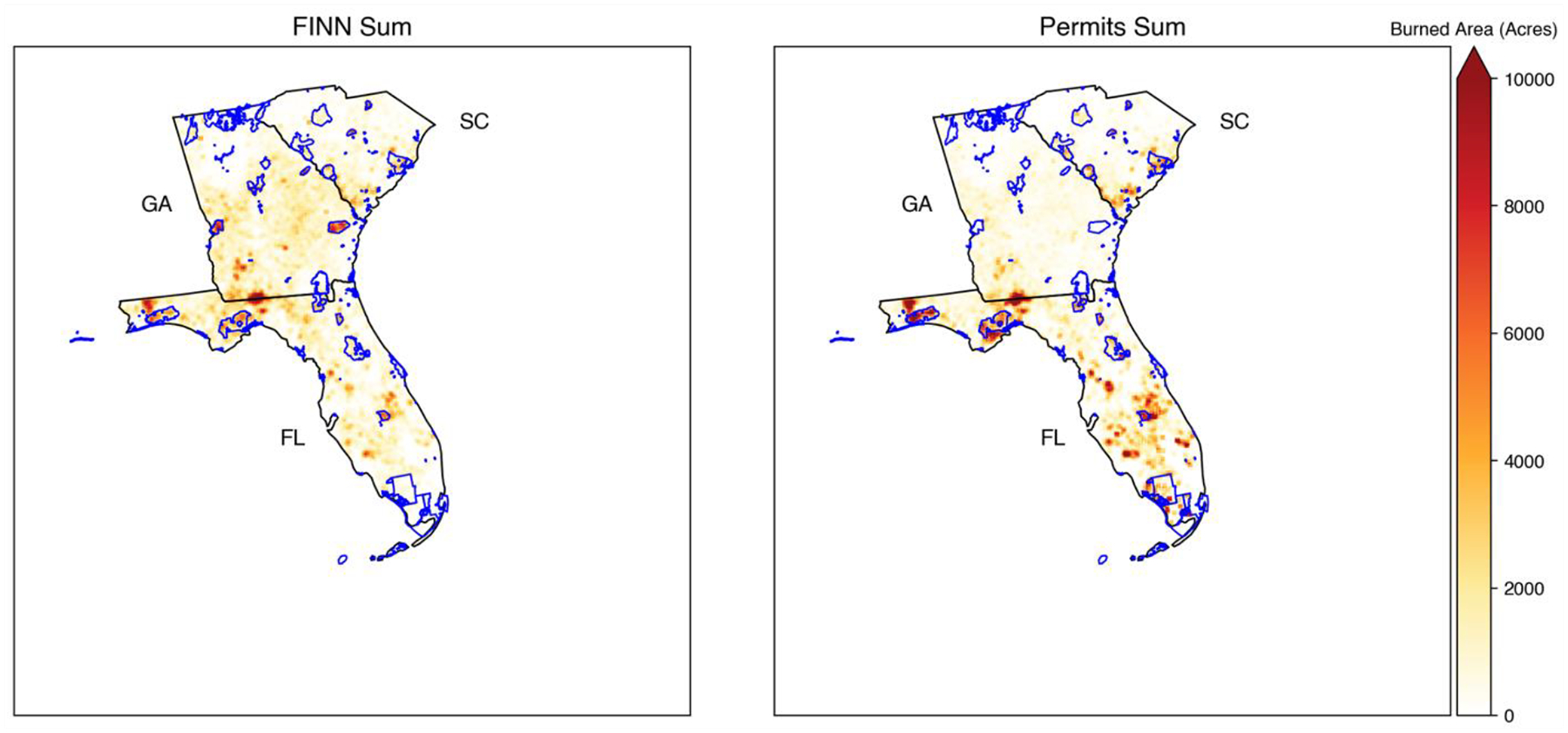
The total grid-based burned area from FINN and permit. FINN covered prescribed burns from 2013 to 2020. Georgia permits included records from 2015 to 2020. South Carolina and Florida permits included records from 2013 to 2020. Federal land boundaries are shown in blue. (State abbreviations: GA: Georgia; FL: Florida; SC: South Carolina).

**Figure 5.**
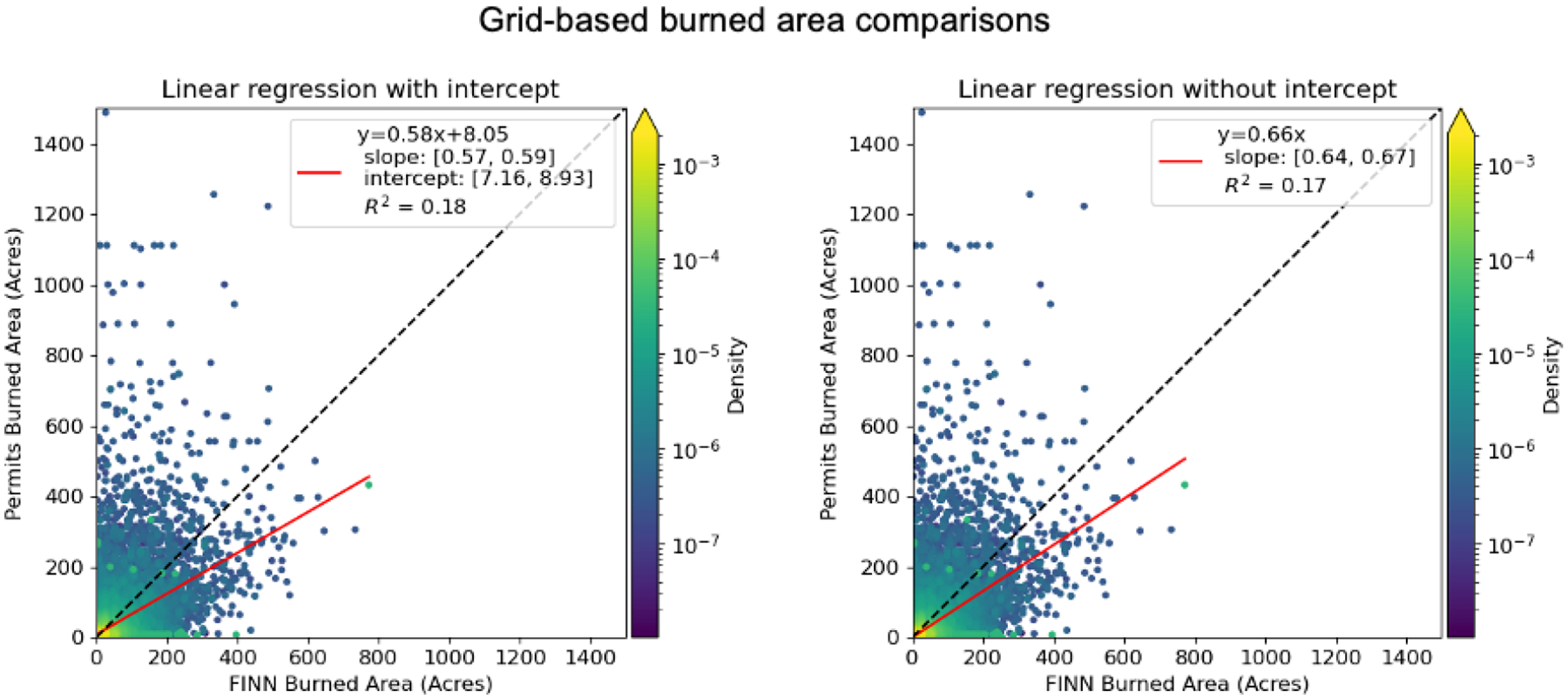
A linear regression between the FINN burned area and permitted burn area (unit: acres) of prescribed burns matched over 4 km grid cells in Florida, Georgia, and South Carolina. The black line is a 1:1 line and the red line is the regression line. Uncertainty of linear regression parameters was reported with a 95% confidence interval.

The “segmentation” of fires where FINN may detect several fire points for a single prescribed burning event, especially for larger burns, can help to explain why the matching performance is sensitive to spatial resolution. The burned area detected by FINN should be calculated by summing all burned areas from all fire points related to a specific fire event. Grid-based burned area matching was considered since it can partly mitigate the issues due to the segmentation of FINN and the uncertainty of FINN or permit locations. Additionally, grid-based emissions, which are derived from the grid-based area, can still be utilized in chemical transport models. We aggregated point-wise FINN and permit records to a 4 km grid definition. Burns that were detected from both FINN and permits at the same date and the same grid were considered in the grid-based matching. Additionally, a sampling method that used an average value of 3 by 3 grids to represent the value of the center grid was employed to mitigate the impact of different grid definitions. The grid-based burned areas from FINN and permits had a similar spatial pattern in Florida, South Carolina, and Georgia (Figures 4 and S9). In Georgia, burns in some federal lands were missing in permit records since federal burners were not required to apply to Georgia Forestry Commission for permits; therefore, those lands were excluded from our analysis. By comparing the matched grid-based burned area from FINN with permits, the R^2^ was higher than 0.17, which was much better than the fire-to-fire matching (Figure 5). The slope of the linear regression model was less than 1, which indicated FINN overestimated the burned area of prescribed burns in these selected states. Additionally, a linear regression with an intercept showed a positive offset, which means FINN underestimated the burned area of prescribed burns for small fires. Since the performance differences between linear regression with or without the intercept were subtle, we decided to apply the slope of the linear regression model without the intercept (0.66 from Figure 5) as the scaling factor to adjust the southeastern burned area from FINN. The adjusted burned area was utilized in the BlueSky model to estimate prescribed burning emissions.

### 4.3. Prescribed Burning Emissions

FINN estimates fire emissions by burned area and emission factors for different land cover types [43–47]. The data are provided as point emissions, and the heights of emissions are estimated by different plume rise models or assumptions made in different chemical transport models. For example, the Community Multiscale Air Quality Modeling System (CMAQ) [48] uses the Briggs plume rise model [49] for point sources. WRF-Chem [50] uses a 1D plume rise model proposed by Freitas et al. [51] to estimate the injection height of emissions. The public version of the Goddard Earth Observing System-Chem (GEOS-Chem) [52] assumes that all biomass-burning emissions are emitted into the atmospheric boundary layer. On the other hand, BlueSky provides different options to estimate the fuel type, fuel load, fuel moisture, and emission factors, which are all determinants of the magnitude of emissions. As for the vertical structure of emissions, BlueSky uses the plume rise models that it incorporates such as FEPS [36], Briggs [49], and Sofiev [53]. FEPS with Briggs plume top behaviors is utilized in this study to provide plume height for the three-dimensional prescribed burning emissions. Fire activity information such as start time, end time, location, and burned area is required. In this study, we used the original FINN burned area to run BlueSky and compared the differences between emission estimation methods from FINN and BlueSky. We also compared prescribed burning emissions from adjusted FINN burned areas and permits to understand the discrepancies between satellite-derived data and ground-based data, and evaluated the uncertainty associated with our estimations of emissions from prescribed burning.

#### 4.3.1. Emission Comparison between FINN and BlueSky

**Figure 6.**
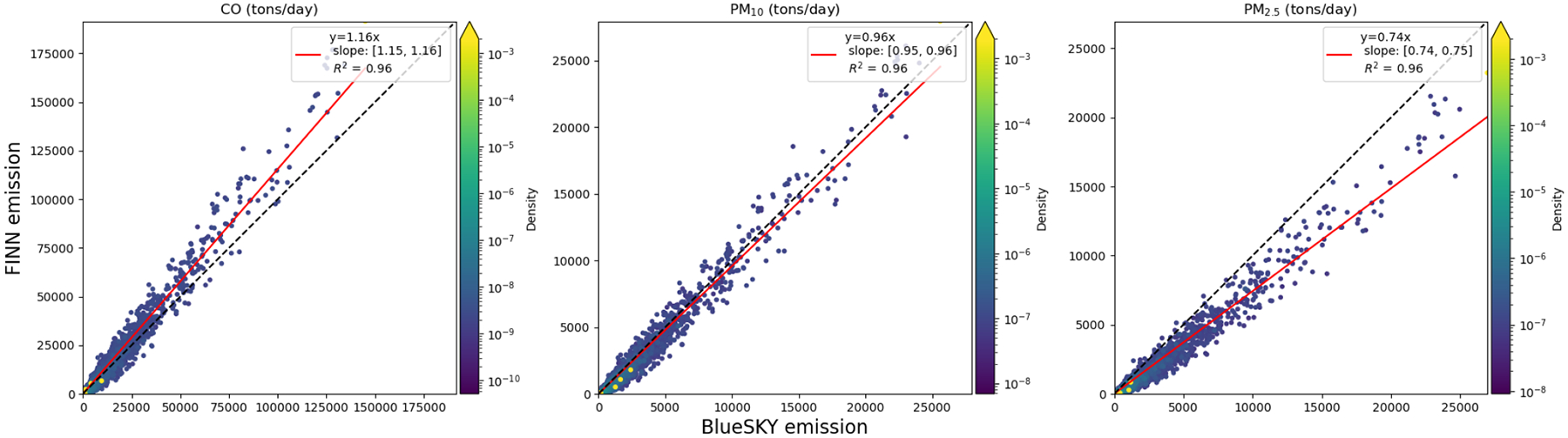
A comparison between estimated FINN and BlueSky daily total prescribed burning emissions in the southeastern United States. The black line is a 1:1 line and the red line is the regression line. Uncertainty of the linear regression parameters was reported with a 95% confidence interval.

The daily total emissions for the southeastern United States were compared between FINN and BlueSky. A linear regression without an intercept was conducted to understand the agreement between FINN and BlueSky emissions (Figure 6). Particulate matter and CO estimated from the two different methods were highly correlated (R^2^ = 0.96). PM_2.5_ and PM_10_ emissions from FINN were 74% and 96% of the respective BlueSky emissions. CO emissions from FINN were 16% higher than BlueSky CO emissions.

**Figure 7.**
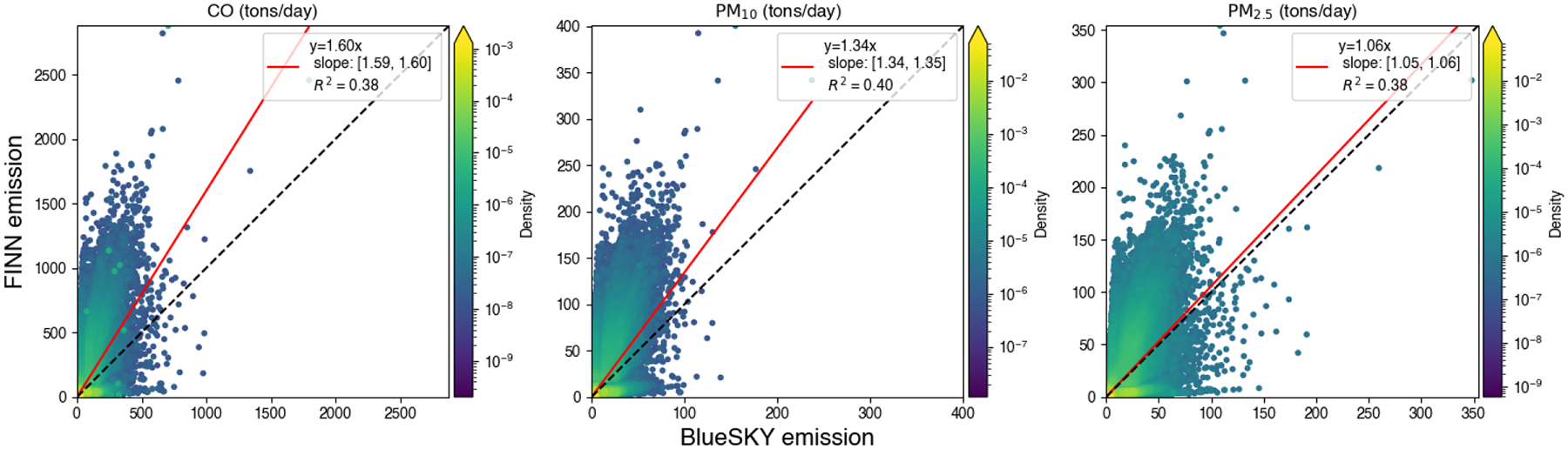
A comparison between estimated FINN and BlueSky prescribed burning emissions for each record in FINN. The black line is a 1:1 line and the red line is the regression line. Uncertainty of the linear regression parameters was reported with a 95% confidence interval.

Although daily total emissions from FINN and BlueSky were consistent with each other, a comparison of emissions for individual fires showed discrepancies. FINN statistically has higher CO and particulate matter emissions from a single fire event compared to the BlueSky framework (Figure 7).

**Figure 8.**
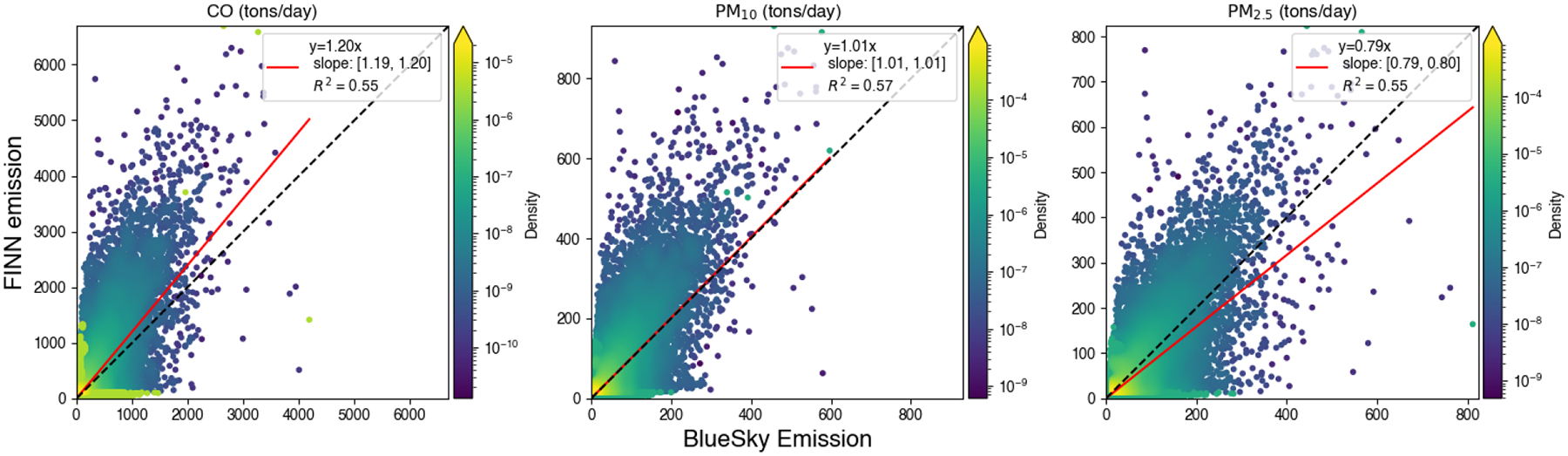
A statewide comparison between estimated FINN and BlueSky prescribed burning emissions under 4 km grid definition. The black line is a 1:1 line and the red line is the regression line. Uncertainty of the linear regression parameters was reported with a 95% confidence interval.

For regional chemical transport model simulations, typical grid-based emissions are provided for the model. We compared the FINN and BlueSky emissions under a 4 km grid definition to understand the differences in emission inputs (Figure 8). The R^2^ between estimated FINN and BlueSky emissions was higher than 0.55. FINN had higher CO and lower PM_2.5_ emissions than the BlueSky method, while PM_10_ emissions were close.

#### 4.3.2. Prescribed Burning Emissions from Adjusted FINN Burned Area and Permits

In this study, we developed two different sets of grid-based emissions. One is generated using burned area from permits (Figures S10–S12), and the other is generated employing the adjusted FINN burned area (Figures S13–S15). The magnitude and vertical structure of the emissions were estimated using BlueSky.

**Figure 9.**
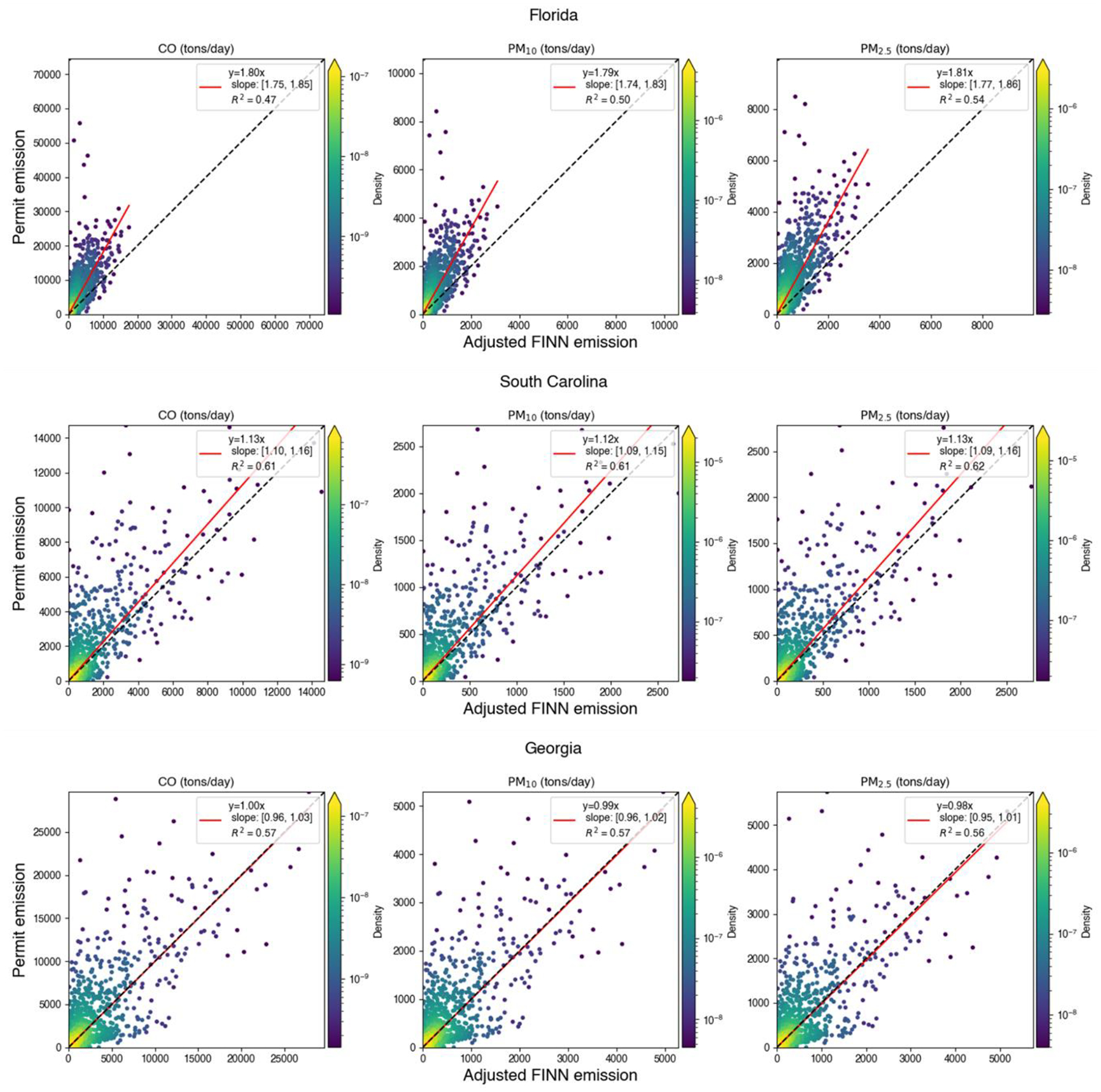
A comparison between permit and adjusted FINN daily total prescribed burning emissions in Florida, South Carolina, and Georgia. The black line is a 1:1 line and the red line is the regression line. Uncertainty of the linear regression parameters is reported with a 95% confidence interval.

To understand the differences in daily budgets of the emissions from permit and adjusted FINN burned areas, we compared the daily total emissions of prescribed burning in these three states (Figure 9).

**Figure 10.**
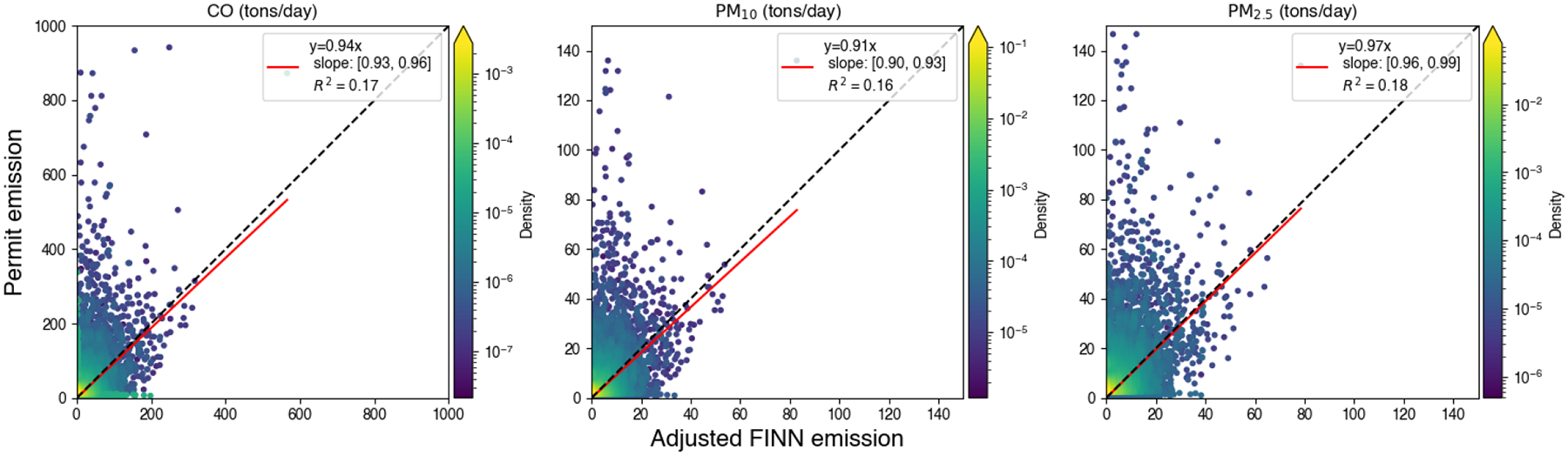
A comparison between permit and adjusted FINN-prescribed burning emissions in matched grid cells under a 4 km grid definition. The black line is a 1:1 line and the red line is the regression line. Uncertainty of the linear regression parameters was reported with a 95% confidence interval.

We also conducted a grid-based comparison between adjusted FINN and permit emissions in matched grid cells (Figure 10) based on the grid-based matching algorithm. The R^2^ between the permit and adjusted FINN grid-based emissions were 0.17, 0.16, and 0.18 for CO, PM_10_, and PM_2.5_, respectively, which were close to the correlation for the grid-based burned area above (R^2^ = 0.17).

#### 4.3.3. Emissions Comparison with NEI

To evaluate FINN and its adjusted version, we compared its emissions with those in the National Emissions Inventory (NEI) for 2014 [54] and 2017 [55] (Figures S16–S21). Specifically, we compared emissions from different sectors, including agricultural burning, wildfire, and prescribed burning, using both NEI and FINN data processed through our burn-type differentiation algorithms. The result reveals that prescribed burning was the main source of emissions from wildland fires in the southeastern United States, with the exception of Virginia (VA) and West Virginia (WV) in 2014, where wildfires were dominant. Furthermore, our results show that FINN had higher total emissions than the NEI in most states. This is in contrast to Larkin et al. [22], who reported that the NEI in 2014, which included GOES and MODIS, had higher fire emissions than version 1.5 of FINN (MODIS only). This difference can be explained by the utilization of VIIRS in FINN version 2.5, which has higher spatial resolution than MODIS and can detect more prescribed burns. For our adjusted FINN, prescribed fire emissions were lower than FINN due to a 34% reduction in the burned area but they were still higher than the NEI in most states.

## 5. Discussion

Burn permits and satellite products are the main resources for fire activity information necessary for prescribed burning emissions estimation. Burn permits provide prescribed burning records but are only available in some states. Satellite products detect worldwide wildland fires but detecting small fires can be particularly challenging. Additionally, satellite products do not differentiate the burn types of fires. Some studies [20,21] assume fires detected by satellite products are all prescribed burnings in the southeastern United States because prescribed burning is more common than the other types of fires. The method for burn-type differentiation is still essential for prescribed burning emissions estimation from satellite products since wildfires and prescribed burning have different fire behaviors and emission patterns [7]. Prescribed burning emissions estimation also relies on the burned area and emission factors. So, we derived an adjustment factor for the FINN burned area and evaluated the uncertainty of the emissions across different frameworks (FINN-provided emissions and BlueSky).

### 5.1. Burn-Type Differentiation

When evaluating the efficiency of our algorithm for detecting agricultural burns, the disparity between permit-provided and NLCD-based burn types can be explained by the uncertainties in NLCD data and the location and burn area of permit data. Although NLCD updates the land cover data every three years, there is still a two-year gap between each update. The changes in Florida’s cropland percentage and spatial extent are relatively large, as indicated by Auch et al. [56]. Meanwhile, NLCD, which is derived from multiple satellite imageries, has uncertainties in land type classifications. Wickham et al. [57] evaluated the uncertainty of NLCD 2016 and reported an 86.4% and 90.6% overall accuracy for level II and level I data, respectively. For reported permit data, the land cover type may not be accurate if the recorded location is not accurate. Additionally, the square burned area assumed in the algorithm could include other land types than the actual burned area, which would change the dominant land cover type for the fire.

In the wildfire detection algorithm, the disparity between the WFIGS and our algorithm can be explained for several reasons. First, WFIGS reports 9460 wildfires (without filtering out small wildfires) in the southeastern United States, which is much lower than the number of fires (130, 780) reported in the national statistics of wildfires provided by the National Interagency Fire Center [58], indicating that WFIGS is not a complete dataset for wildfires. Meanwhile, prescribed burns in neighboring lands conducted on consecutive days can be misclassified as wildfires by the wildfire detection algorithm. Additionally, some wildfires are missed in FINN due to cloud cover or other obstacles, such as the tree canopy [59]. FINN without burn-type differentiation only matches 53.21% of wildfires in WFIGS. Additionally, the fires that cannot be retrieved by FINN may affect the estimation of fire duration, leading to misclassification. For example, a three-day wildfire detected on the first and last days will be falsely classified as two separate prescribed burns.

### 5.2. Matching Prescribed Burning Records in FINN with Permits

The correlations between FINN burned area estimates and permit estimates vary under different spatial resolutions. They can be partly explained by the spatial segmentation of FINN. Although FINN clusters FRP from VIIRS and MODIS, it is still challenging to decide whether several records which are near to each other belong to the same burning event. Our spatial clustering analysis shows FINN separates fires that have a distance larger than 300 m (Figure S1), and it is possible that the records at such close distance belong to the same event.

### 5.3. Prescribed Burning Emissions

The prescribed burning emission estimations can differ from different methods (FINN or BlueSky) or fire activity data sources (adjusted FINN or permits). Prescribed burning emissions estimated by FINN or BlueSky have a high correlation for southeastern daily total emissions and a low correlation when making a fire event comparison. This result indicates that evaluating specific prescribed burning impacts on air quality would yield different results when the FINN or BlueSky method is employed. In this study, we used BlueSky to provide the prescribed burning emissions for the southeastern United States since the estimation of prescribed burning emissions in the CONUS (contiguous United States) from BlueSky differs from FINN in several ways. Firstly, BlueSky uses FCCS, which includes more detailed fuel types than FINN. Additionally, BlueSky separates wildfire and prescribed burning for emission calculation and integrates emission factors from laboratory or field studies concentrated in the United States [35], while FINN concentrates on global emission estimation and does not treat wildfire and prescribed burning differently. Moreover, BlueSky estimates fuel moisture, fuel consumption, and plume height by considering meteorological conditions. This information affects the magnitude and vertical structure of emissions. Meanwhile, FINN does not need meteorological conditions for emission estimation.

Prescribed burning emission comparisons between adjusted FINN and permits show a similar correlation as burned area comparisons, which indicates that burned area estimation is important in prescribed burning emission estimations. For daily total emissions, adjusted FINN emissions are close to permit-based emissions in Georgia and South Carolina after applying the adjustment factor for the burned area. On the other hand, permit emissions are about 80% higher than adjusted FINN emissions in Florida. The reason for the low adjusted FINN emissions in Florida is that we developed the scaling factor to reduce FINN burned area based on all three states (Figure 3). A burned area comparison for Florida indicates a scaling factor to increase the burned area, which is a different pattern among these three states. For grid-based emission comparisons, the slope of the linear regression without the intercept is close to one since we adjusted burned area before BlueSky modeling. Without the burned area adjustment, the same slope was only 0.6 (Figure S24). This indicates that the FINN burned area adjustment mitigates the difference between FINN-based and permit-based emissions. This also implies that the adjustment factor derived from the slope of linear regression is robust, even though the R^2^ of the linear regression between permit and FINN burned areas was low.

## 6. Conclusions

Wildland fires identified from the satellite-derived product FINN have been segregated into different burn types (prescribed, agricultural burns, or wildfires) by considering the land cover type of fire locations and fire durations. The burned area of prescribed burns using FINN-based wildland fire estimates is compared with the burned area of prescribed burning permits in Georgia, Florida, and South Carolina. Matching and comparisons between the burned area from FINN and permits are conducted at various resolutions. FINN burned area estimates have a low correlation (R^2^ < 0.05) with permit estimates based on fire-to-fire matching, while the correlation is relatively stronger for grid-based (R^2^ > 0.17) and statewide (R^2^ > 0.55) matching. A linear regression model, developed using grid-based matching results, determined that the prescribed burned area from FINN needs to be reduced by 34% of the FINN burned area. Using the BlueSky framework with the adjusted burned area, prescribed burning emissions are estimated for the southeastern United States from 2013 to 2020. To understand the emission differences between FINN and BlueSky, we also ran BlueSky with unadjusted FINN burned areas. The comparisons between emissions from FINN and BlueSky indicate that the differences in emissions can be large for single fire events (R^2^ > 0.38), but much smaller when considering emission estimates at a 4 km grid resolution (R^2^ > 0.55), or when assessing statewide emissions (R^2^ > 0.96). We also compare emissions estimated from permit burn areas and the adjusted FINN burned area using BlueSky to understand the uncertainty of prescribed burning emissions stemming from the potential use of different data sources. The linear regression model between adjusted FINN and permit emissions has a slope of around 0.94 with R^2^ = 0.17. The result of this comparison indicates that the magnitude of emissions from the adjusted FINN burned area at a 4 km grid resolution agrees with those derived from permit burn areas. The methods we presented here are readily useable for burn-type differentiation, matching and comparison of the burned area between two datasets under various resolutions, and estimation of prescribed burning emissions. This study also benefits health studies related to prescribed burning since type-differentiated and more accurately estimated prescribed burning emissions from satellite products are needed to model the air quality impacts of prescribed fires. Those air quality impacts are essential to smoke exposure evaluation, which can be used for public health research and surveillance. The emissions data we produced can be readily used for air quality simulations and investigations of the health impacts of prescribed burning in the southeastern United States between 2013 and 2020.

To improve the algorithms and emission products in this study, two main efforts should be considered. Firstly, the state-level burn permit data systems should be unified since it is difficult to obtain data from each state and the data formats vary from one state to another. Burn information in unified permit databases can be grouped into three categories by decreased level of importance. The first group should include latitude, longitude, start date, and burn area, which are the minimum requirements for most fire emission models. This information should be collected when fire managers apply for permits. Updates of burn area data after the burn with the actual burned areas can improve the understanding of the relationships between satellite-derived and ground-based data. The second group may include the start time, end time, and burn type, which can be recorded by the fire managers when they execute the burnings. Start and end times are valuable for generating diurnal time profiles of emissions in chemical transport models. Burn-type information is important for training and evaluating burn-type differentiation algorithms. The third level may include the boundaries of the burned areas, which would be valuable for improving and evaluating clustering algorithms in current fire emission products that combine FRP data from different satellites, such as FINN. Secondly, the burn-type differentiation algorithm can be improved by using statistical models or supervised machine learning models when sufficient reliable wildfire data and prescribed burning permit data are available. The occurrence probability of prescribed burns or wildfires could be related to meteorological conditions or locations, which can be utilized for training data-driven models. Furthermore, the algorithms and frameworks in this study can be applied for agricultural burning and wildfire emissions estimation using corresponding emission factors.

## Supplementary Material

remotesensing-15-02725-v2 Table S1: Duration of prescribed burning; Algorithm S1: Wildfire detection algorithm; Algorithm S2: Nearest distance matching algorithm; Algorithm S3: Relaxation of distance and date matching algorithm; Figure S1: The number of clusters under different distances for clustering for FINN; Figure S2: The number of wildfires (clusters) under different combinations of spatial and temporal clustering distances; Figure S3: Percentage of the records number for different burn types in the southeastern states from FINN, 2013–2020; Figure S4: Statewide matching between FINN and permits in Florida, South Carolina, and Georgia; Figure S5: Daily total burned area in Florida, South Carolina, and Georgia; Figure S6: Matching performance under different selected distance thresholds; Figure S7: Matching performance under different combinations of relaxation date and relaxation distance; Figure S8: The number of matched pairs between FINN and permits under different combinations of relaxation date and relaxation distance; Figure S9: Total grid-based burned area of prescribed burnings from FINN and permit for 2013–2020; Figure S10: Yearly permit-based total CO emission in South Carolina, Georgia, and Florida from 2013–2020; Figure S11: Yearly permit-based total PM_2.5_ emission in South Carolina, Georgia, and Florida from 2013–2020; Figure S12: Yearly permit-based total PM_10_ emission in South Carolina, Georgia, and Florida from 2013–2020; Figure S13: Yearly adjusted FINN total CO emission in South Carolina, Georgia, and Florida from 2013–2020; Figure S14: Yearly adjusted FINN total PM_2.5_ emission in the southeastern U.S. from 2013–2020; Figure S15: Yearly adjusted-FINN total PM_10_ emission in southeastern U.S. during 2013–2020; Figure S16: PM_2.5_ Emissions comparisons among FINN, adjusted FINN and NEI for 2014; Figure S17: PM_10_ Emissions comparisons among FINN, adjusted FINN and NEI for 2014; Figure S18: CO Emissions comparisons among FINN, adjusted FINN and NEI for 2014; Figure S19: PM_2.5_ Emissions comparisons among FINN, adjusted FINN and NEI for 2017; Figure S20: PM_10_ Emissions comparisons among FINN, adjusted FINN and NEI for 2017; Figure S21: CO Emissions comparisons among FINN, adjusted FINN and NEI for 2017; Figure S22: Breakdown of total burned area from FINN by different burn types in southeastern states, 2013–2020; Figure S23: Breakdown of total burned area from permits by different burn types in Georgia, Florida, and South Carolina; Figure S24: Comparison between permit and BlueSky prescribed burning emissions with unadjusted FINN burned area in matched grid cells under a 4-km grid definition; Figure S25: States in the southeastern United States included in the study.

## Figures and Tables

**Figure 1.**
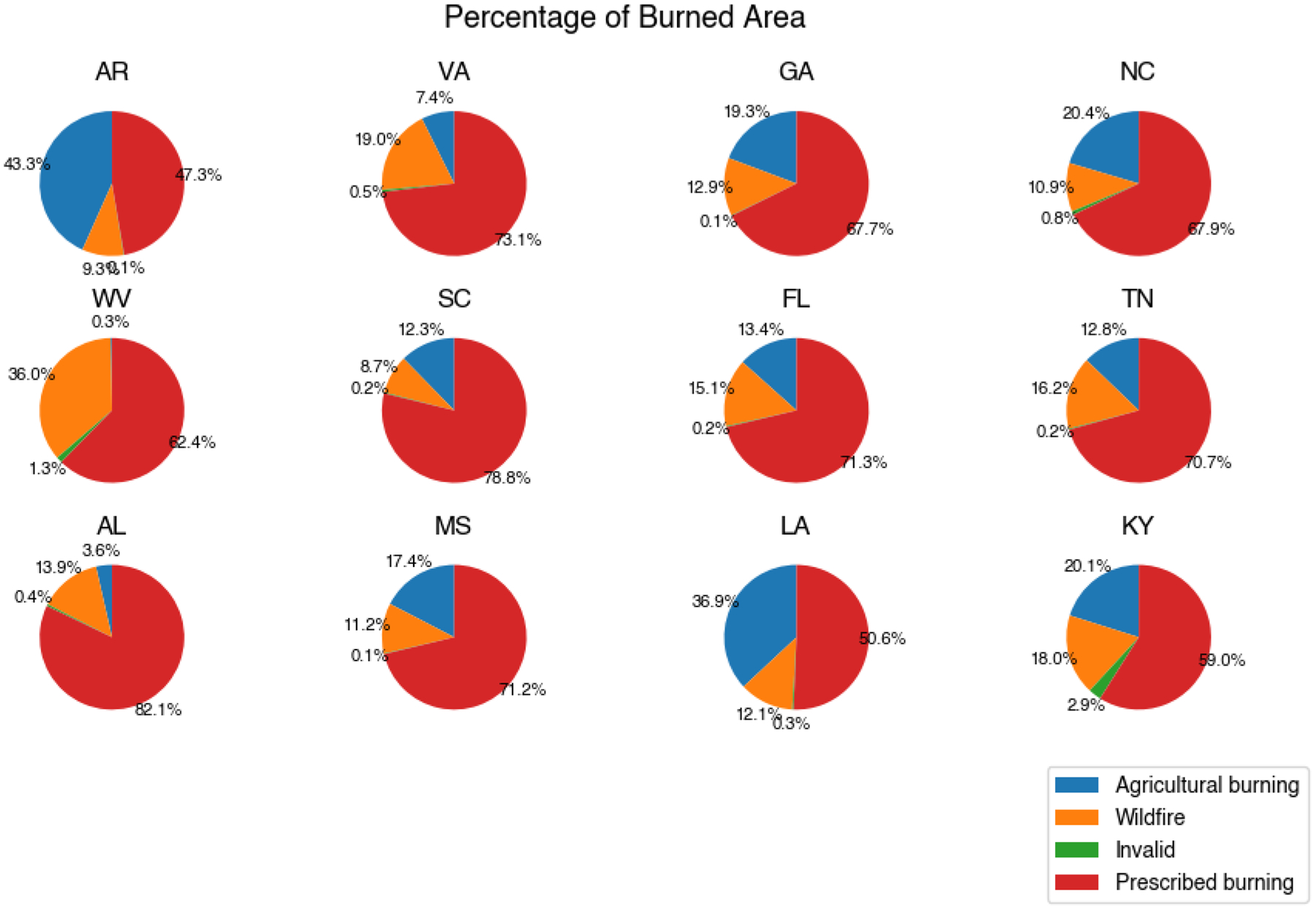
Percentages of the burned area from FINN for different burn types in southeastern states from FINN, 2013–2020. (State abbreviations: AL: Alabama; AR: Arkansas; GA: Georgia; FL: Florida; KY: Kentucky; LA: Louisiana; MS: Mississippi; NC: North Carolina; SC: South Carolina; TN: Tennessee; VA: Virginia; WV: West Virginia).

**Figure 2.**
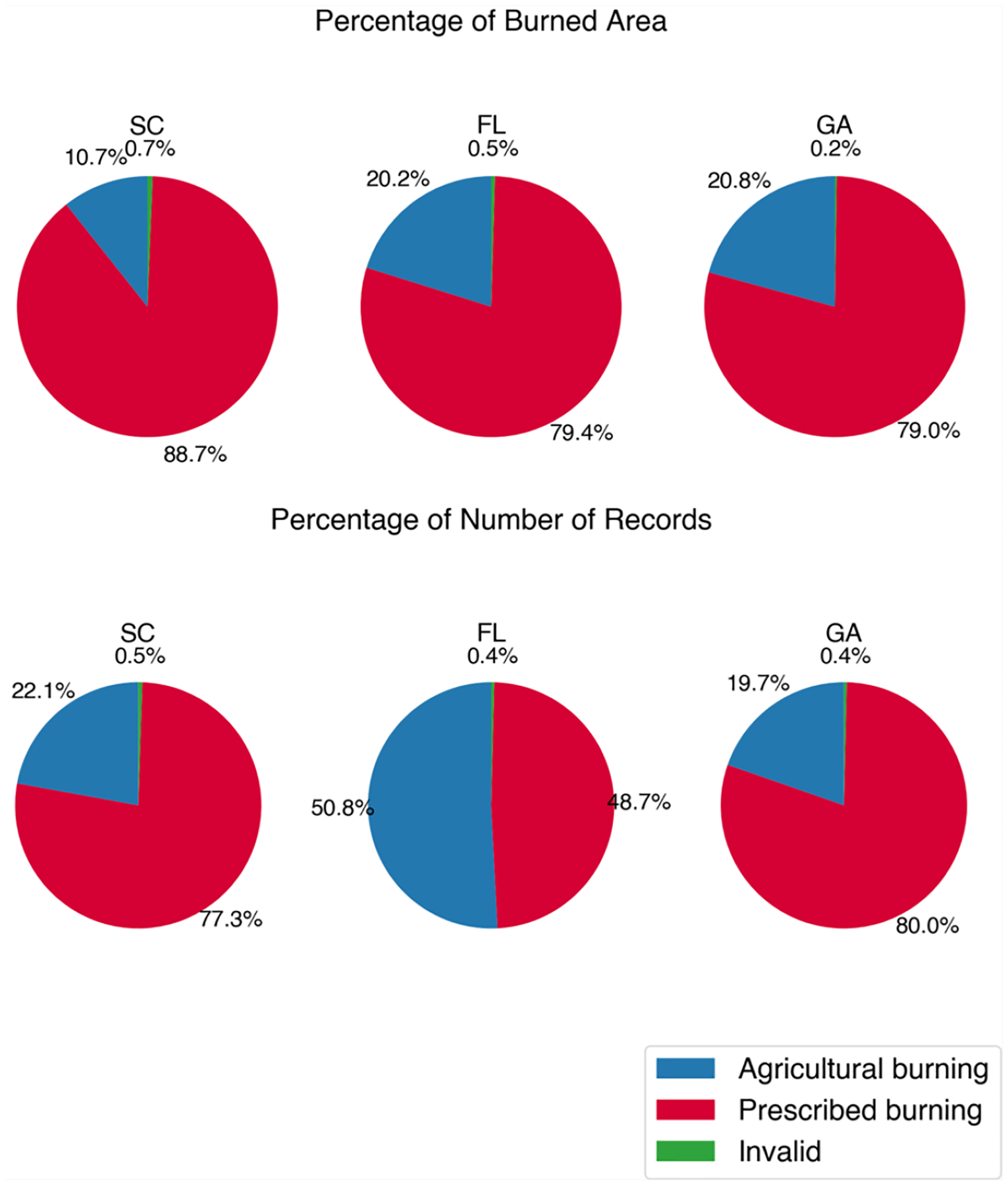
Percentages of the burned area and number of records from permits for different burn types in southeastern states. Georgia permits cover 2015–2020. Florida and South Carolina permits cover 2013–2020. (State abbreviations: GA: Georgia; FL: Florida; SC: South Carolina).

## Data Availability

The code related to this study is available on Github: https://github.com/zli867/RxFireEmission (accessed on 19 May 2023). Data are available in a publicly accessible repository: https://doi.org/10.5281/zenodo.7569933 (accessed on 19 May 2023).
