# Supplementary material for: An Analysis of Prescribed Fire Activities and Emissions in the Southeastern United States from 2013 to 2020
Source: Remote Sens (Basel). Author manuscript; Available in PMC 2024 Aug 2. (PMC11296730; doi:10.3390/rs15112725)
Supplement: remotesensing-15-02725-v2 — Table S1: Duration of prescribed burning; Algorithm S1: Wildfire detection algorithm; Algorithm S2: Nearest distance matching algorithm; Algorithm S3: Relaxation of distance and date matching algorithm; Figure S1: The number of clusters under different distances for clustering for FINN; Figure S2: The number of wildfires (clusters) under different combinations of spatial and temporal clustering distances; Figure S3: Percentage of the records number for different burn types in the southeastern states from FINN, 2013–2020; Figure S4: Statewide matching between FINN and permits in Florida, South Carolina, and Georgia; Figure S5: Daily total burned area in Florida, South Carolina, and Georgia; Figure S6: Matching performance under different selected distance thresholds; Figure S7: Matching performance under different combinations of relaxation date and relaxation distance; Figure S8: The number of matched pairs between FINN and permits under different combinations of relaxation date and relaxation distance; Figure S9: Total grid-based burned area of prescribed burnings from FINN and permit for 2013–2020; Figure S10: Yearly permit-based total CO emission in South Carolina, Georgia, and Florida from 2013–2020; Figure S11: Yearly permit-based total PM2.5 emission in South Carolina, Georgia, and Florida from 2013–2020; Figure S12: Yearly permit-based total PM10 emission in South Carolina, Georgia, and Florida from 2013–2020; Figure S13: Yearly adjusted FINN total CO emission in South Carolina, Georgia, and Florida from 2013–2020; Figure S14: Yearly adjusted FINN total PM2.5 emission in the southeastern U.S. from 2013–2020; Figure S15: Yearly adjusted-FINN total PM10 emission in southeastern U.S. during 2013–2020; Figure S16: PM2.5 Emissions comparisons among FINN, adjusted FINN and NEI for 2014; Figure S17: PM10 Emissions comparisons among FINN, adjusted FINN and NEI for 2014; Figure S18: CO Emissions comparisons among FINN, adjusted FINN and NEI for 2014; Figure S19: PM2.5 [file NIHMS2012318-supplement-remotesensing-15-02725-v2.zip › remotesensing-2386704-supplementary.pdf]

Supplemental Material for

An Analysis of Prescribed Fire Activities and Emissions in the Southeastern United States from 2013 to 2020

Zongrun Li [1], Kamal J. Maji [1], Yongtao Hu [1], Ambarish Vaidyanathan [2], Susan M. O'Neill [3], M. Talat Odman [1,*] and Armistead G. Russell [1]

[1] School of Civil and Environmental Engineering, Georgia Institute of Technology, Atlanta, GA 30332, USA
[2] National Center for Environmental Health, Centers for Disease Control and Prevention, Atlanta, GA 30341, USA
[3] United States Department of Agriculture Forest Service, Pacific Northwest Research Station, Seattle, WA 98103, USA
* Correspondence: talat.odman@ce.gatech.edu

Contents of this file

Figures 25

Tables 1

Algorithms 3

Table S1: Duration of prescribed burning

Burned Area (A) unit: acres	Duration unit: hour
$A \leq 10$	2
$10 < A \leq 30$	3
$30 < A \leq 70$	4
$70 < A \leq 150$	5
$150 < A \leq 300$	6

300 < A	7

Algorithm S1:

Algorithm 1 Wildfire detection algorithm

Input: FINN fire records

Output: wildfire records in FINN

1: Decide a spatial clustering distance threshold d_s and a temporal clustering distance threshold d_t

2: For each fire f_m in day $i+1$, find the fire record f_{m+1} in day i which has the nearest distance to the fire selected in day $i+1$. If the distance is smaller than d_t, we add an edge $e = \{f_m, f_{m+1}\}$. These connected nodes are belong to a same temporal cluster T_k.

3: For each cluster $T_k = \{e_{k_1}, e_{k_2}, \ldots, e_{k_n}\}$, search all nodes the cluster. The fires are temporally clustered.

4: For each fire f_m in day i, find all fire records f_p $(n = 1, 2, 3 \ldots n)$ in the same day which has the distance less than d_s to the fire selected in day i. We add edges between f_m and f_p $(n = 1, 2, 3 \ldots n)$. These connected nodes are belong to a same spatial cluster S_k.

5: For each cluster $S_k = \{e_{k_1}, e_{k_2}, \ldots, e_{k_n}\}$, search all nodes in the cluster. The fires are spatially clustered.

6: For each fire, if the fire belong to S_m and T_n, add edge between S_m and T_n. Notice that spatial clusters and temporal clusters are the two parts of a bipartite graph which means no edges $e = \{\forall m, n | \ T_m, T_n\}$ and no edges $e = \{\forall m, n | \ S_m, S_n\}$. These connected clusters are belong to a wildfire cluster W_t.

7: Search all temporal and spatial clusters connected in each wildfire cluster W_t. All nodes in these temporal clusters and spatial clusters are considered belong to a same wildfire.

Algorithm S2:

Algorithm 2 Nearest distance matching algorithm

Input: FINN fire records, permit fire records

Output: matched pairs from FINN and permit

1: For each day, find the nearest FINN data for each permit record. The matched pairs are denoted as $P_1 = (permit_i, FINN_j)$.

2: For each day, find the nearest permit record for each FINN data. The matched pairs are denoted as $P_2 = (permit_m, FINN_n)$.

3: Find the intersection of the two sets. $P = P1 \cap P2$.

4: Filter out the pairs which FINN and permit have a distance larger than selected distance threshold d_t. The remained pairs are matched pairs.

Algorithm S3:

Algorithm 3 Relaxation of distance and date matching algorithm

Input: FINN fire records, permit fire records

Output: matched pairs from FINN and permit

1: Decide a distance d_r and duration t_r as distance and date relaxation.

2: For each day i, we select the perimit and FINN during $[i - t_r, i + t_r]$. For each permit p_i in the selected period, find all FINN data f_{i_k} $(k = 1, 2, \ldots, n)$ in the same period has a distance with the permit closer than d_r.

3: For each p_i, match the f_{i_m} which has the smallest difference in burned area among f_{i_k} $(k = 1, 2, \ldots, n)$. The matched pairs are denoted as M_1.

4: For each day i, we select the FINN and permit during $[i - t_r, i + t_r]$. For each FINN f_i in the selected period, find all permit data p_{i_k} $(k = 1, 2, \ldots, n)$ in the same period has a distance with the permit closer than d_r.

5: For each f_i, match the p_{i_m} which has the smallest difference in burned area among p_{i_k} $(k = 1, 2, \ldots, n)$. The matched pairs are denoted as M_2.

6: Find the intersection of the two sets. $M = M_1 \cap M_2$. All pairs in set M are the result.

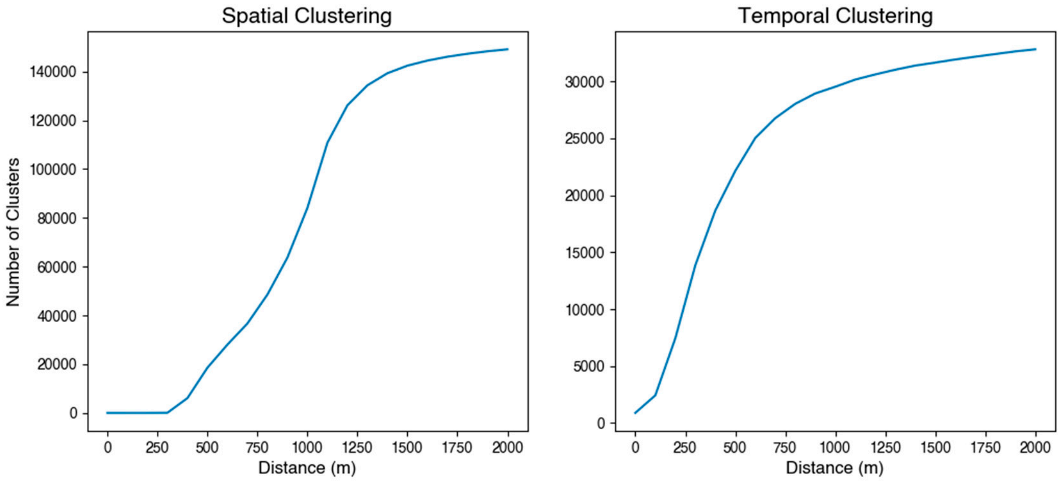

Figure S1. The number of clusters under different distances for clustering for FINN. The left figure is spatial clustering, and the right figure is temporal clustering.

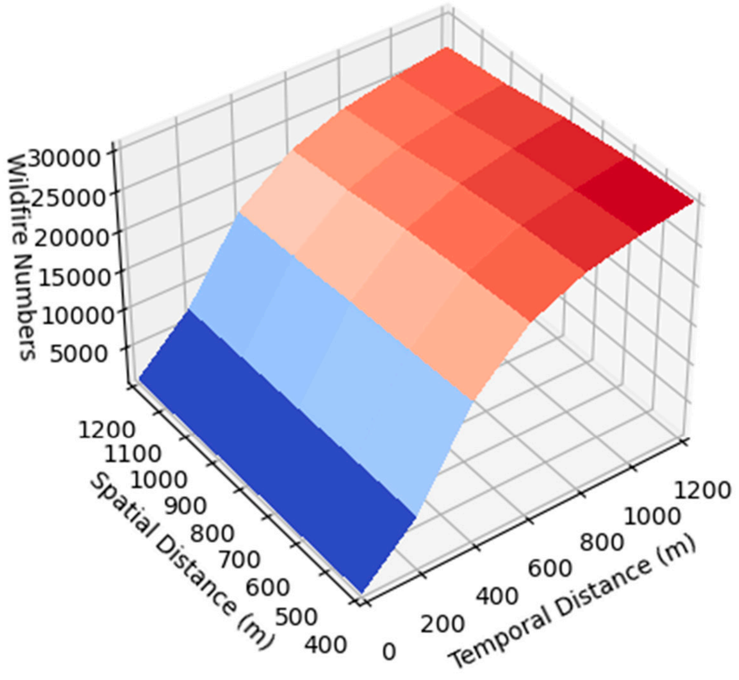

Figure S2. The number of wildfires (clusters) under different combinations of spatial and temporal clustering distances.

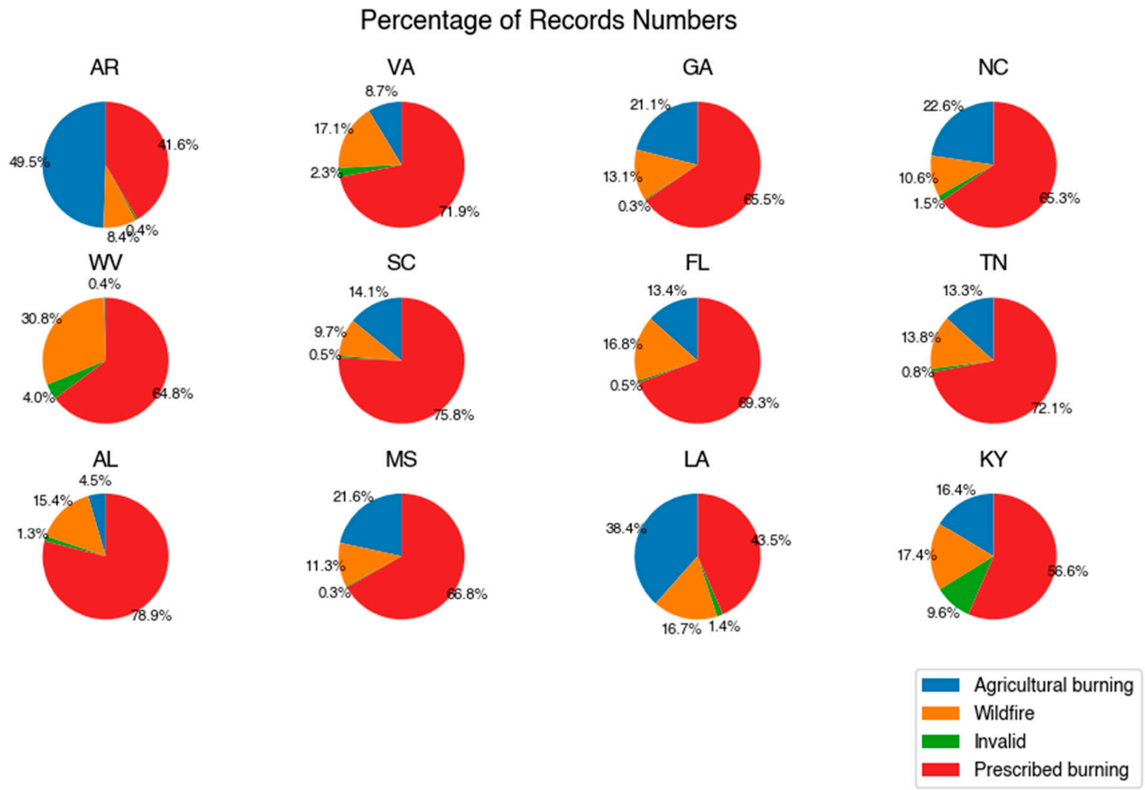

Figure S3. Percentage of the records number for different burn types in the southeastern states from FINN, 2013–2020.

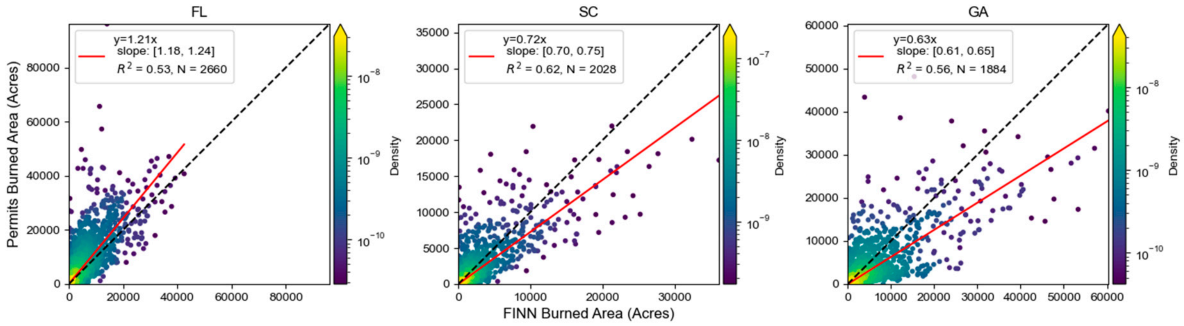

Figure S4. Statewide matching between FINN and permits in Florida, South Carolina, and Georgia. Linear regression without intercept was conducted to fit FINN burned area and permits burned area. The numbers of matching days were indicated as N values. Florida and South Carolina permits cover 2013–2020. Georgia permits cover 2015-2020.

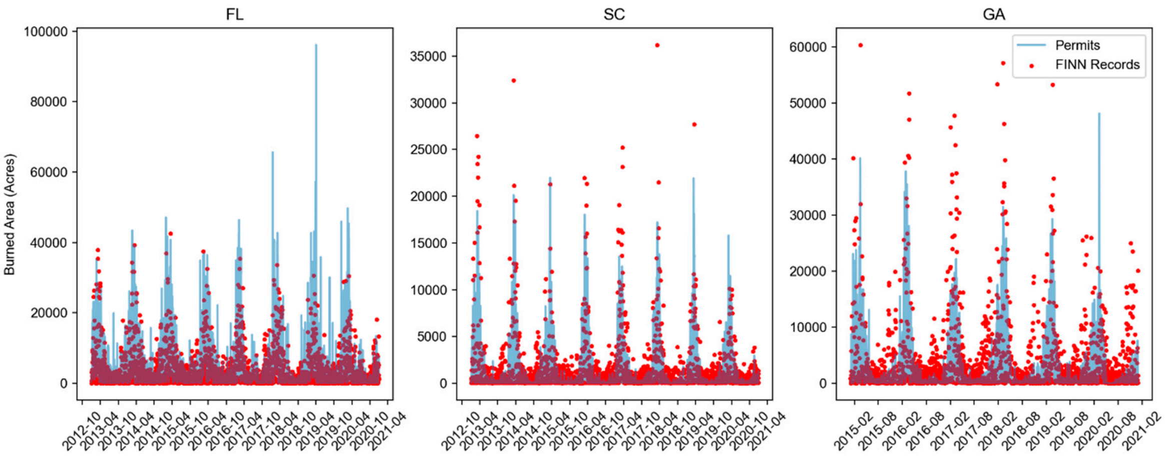

Figure S5. Daily total burned area in Florida, South Carolina, and Georgia. Red scatters are the daily burned area from FINN, and blue lines are the daily burned area from permits.

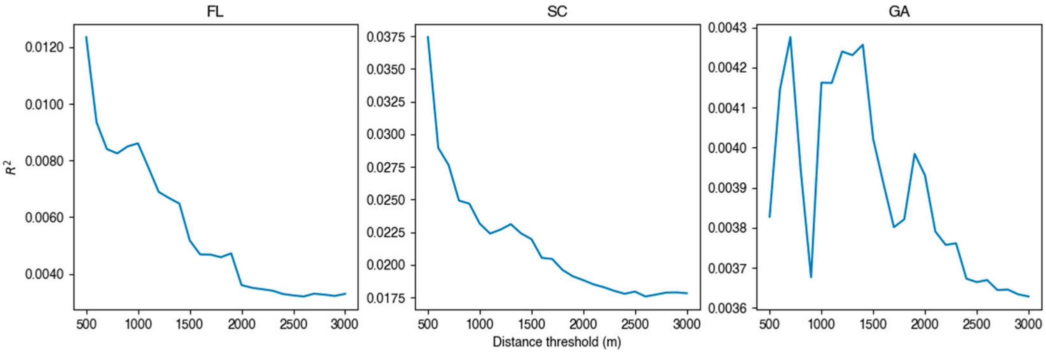

Figure S6. Matching performance (R^2 of the linear regression between matched FINN and permits) under different selected distance thresholds.

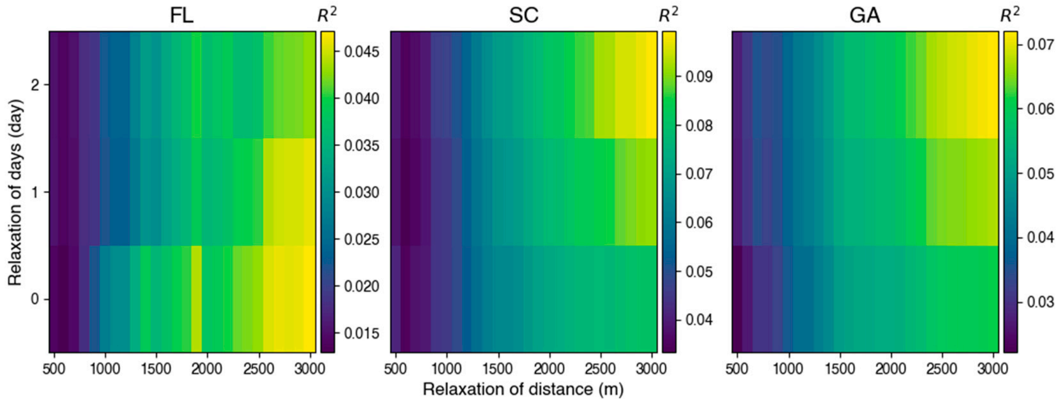

Figure S7. Matching performance (R^2 of the linear regression between matched FINN and permits) under different combinations of relaxation date and relaxation distance.

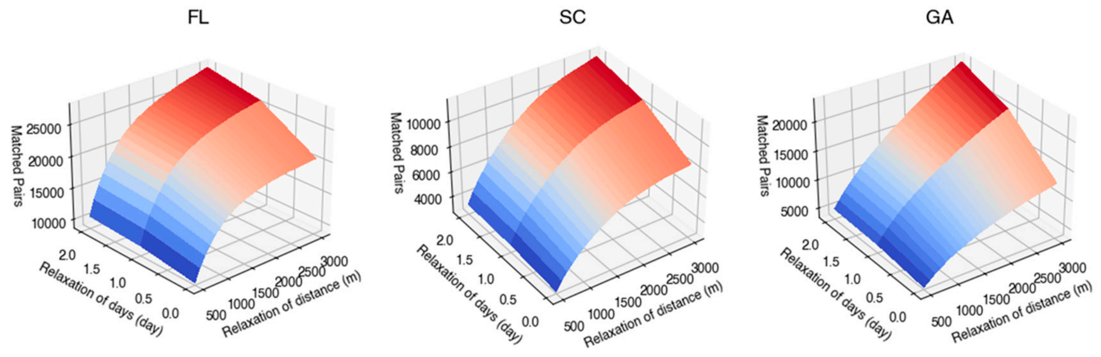

Figure S8. The number of matched pairs between FINN and permits under different combinations of relaxation date and relaxation distance.

For each permit p_i, more relaxations on days and/or distance increase the number of FINN records candidates f_i for matching in Algorithm S3. The difference in the burned area decreases since the algorithm matches the FINN record, in which burned area is the closest to permit burned area among candidates. It explains the higher R^2 when more relaxations are conducted (Figure S7).

However, we will have more matched pairs between FINN and permits when more relaxations are conducted (Figure S8). Permits that are not detected by FINN are mismatched to FINN records with poor quality (large difference on burned area), and it decreases the R^2 between matched FINN and permits. In Florida, R^2 decreases when the relaxation of days increases. It could be due to more poor-quality pairs being included in Florida than in the other two states.

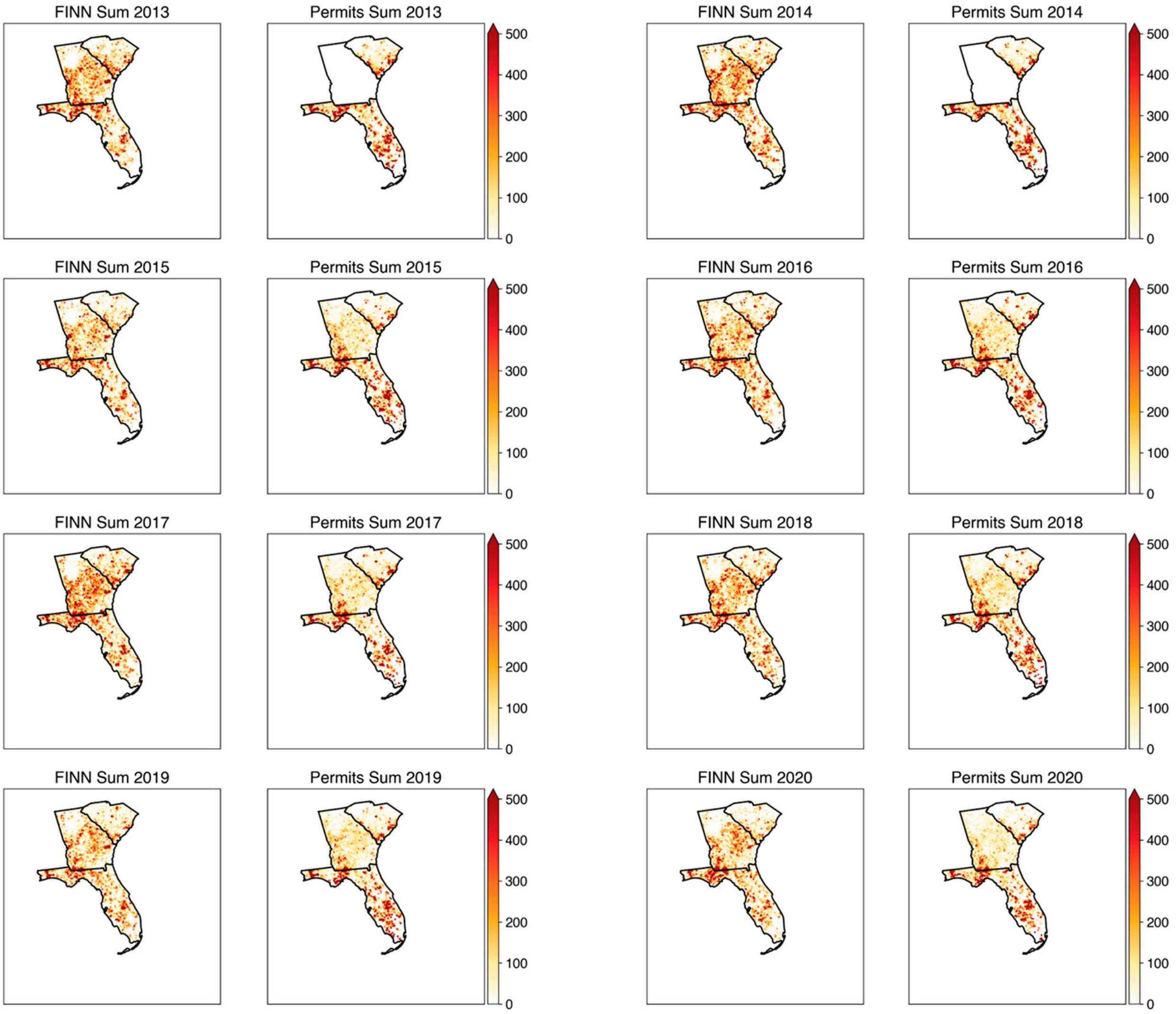

Figure S9. Total grid-based burned area of prescribed burnings from FINN and permit for 2013–2020. Georgia permits data is from 2015 to 2020.

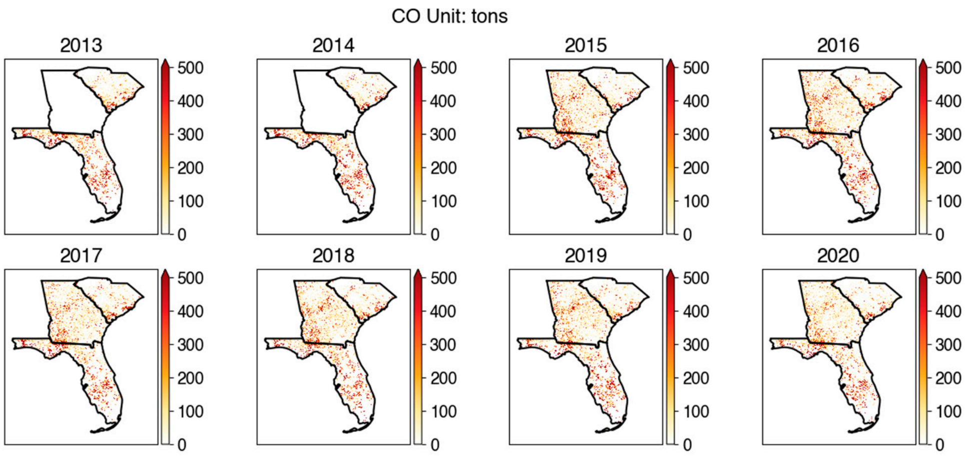

Figure S10. Yearly permit-based total CO emission in South Carolina, Georgia, and Florida from 2013–2020. Permits during 2013–2014 are missing in Georgia.

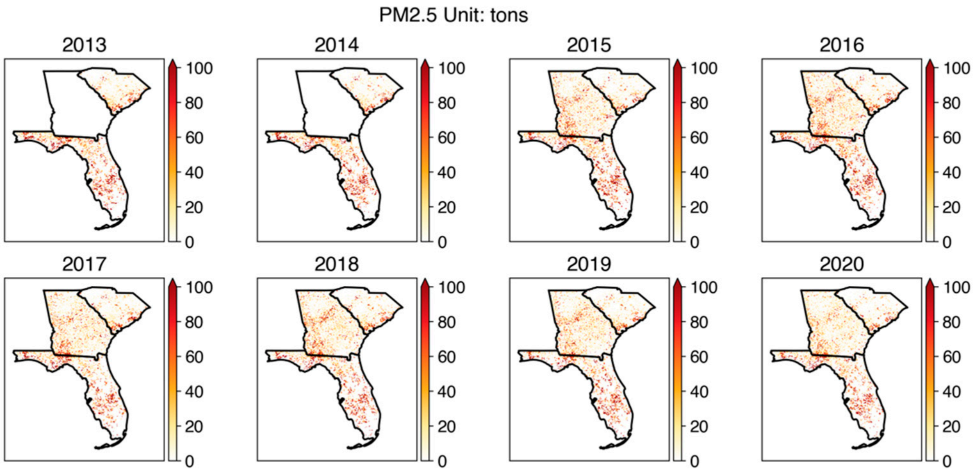

Figure S11. Yearly permit-based total $PM_{2.5}$ emission in South Carolina, Georgia, and Florida from 2013–2020. Permits from 2013–2014 are missing in Georgia.

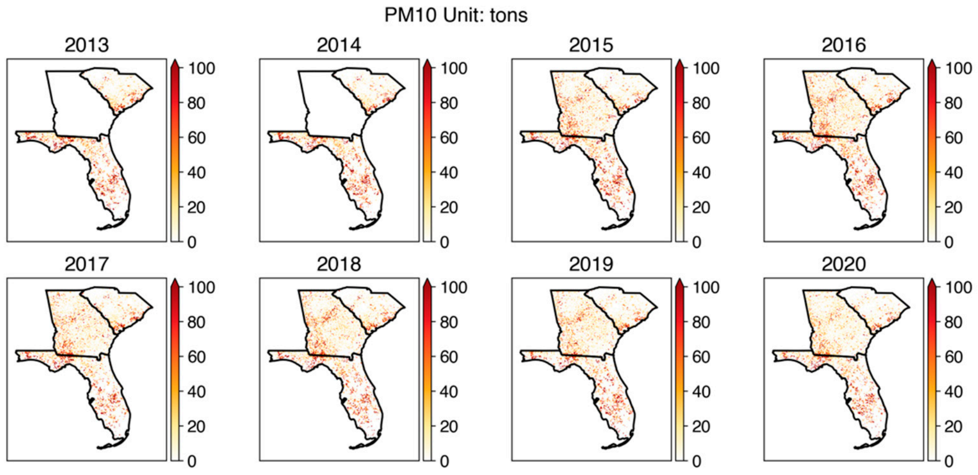

Figure S12. Yearly permit-based total PM_{10} emission in South Carolina, Georgia, and Florida from 2013–2020. Permits from 2013–2014 are missing in Georgia.

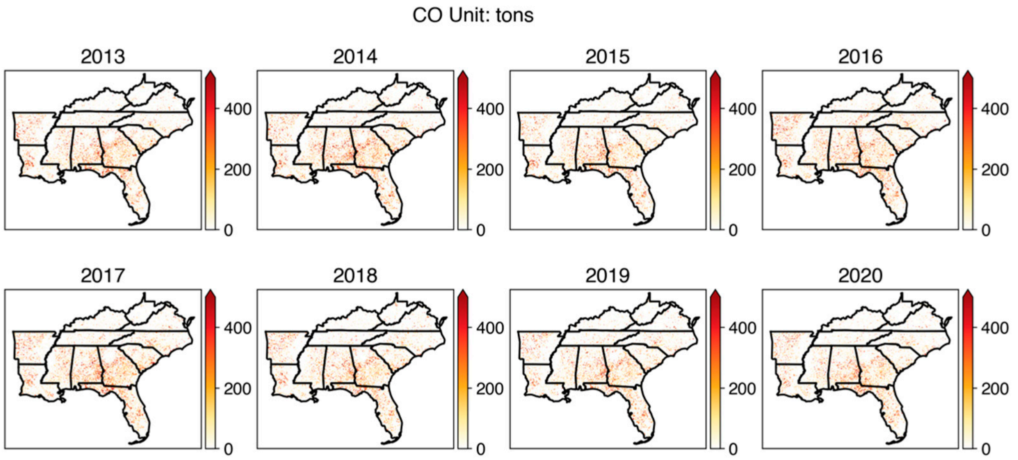

Figure S13. Yearly adjusted FINN total CO emission in South Carolina, Georgia, and Florida from 2013–2020. Permits from 2013–2014 are missing in Georgia.

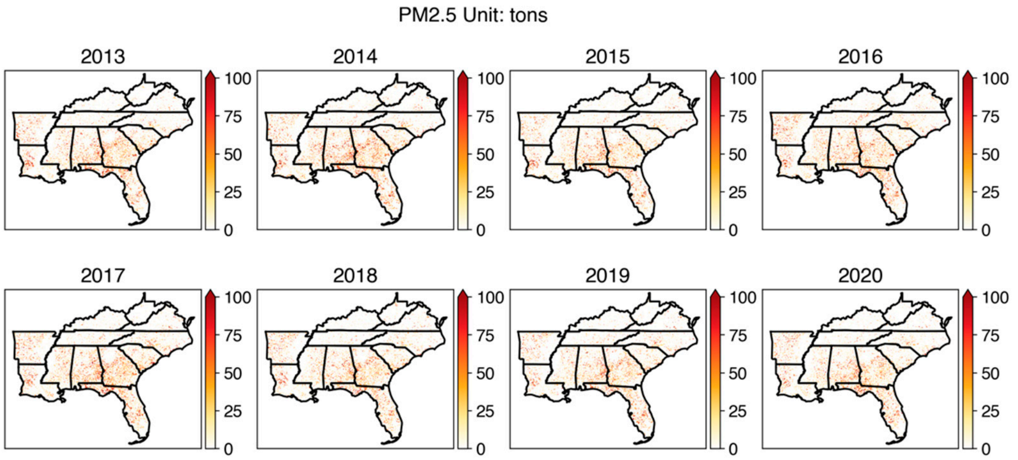

Figure S14. Yearly adjusted FINN total $PM_{2.5}$ emission in the southeastern U.S. from 2013–2020.

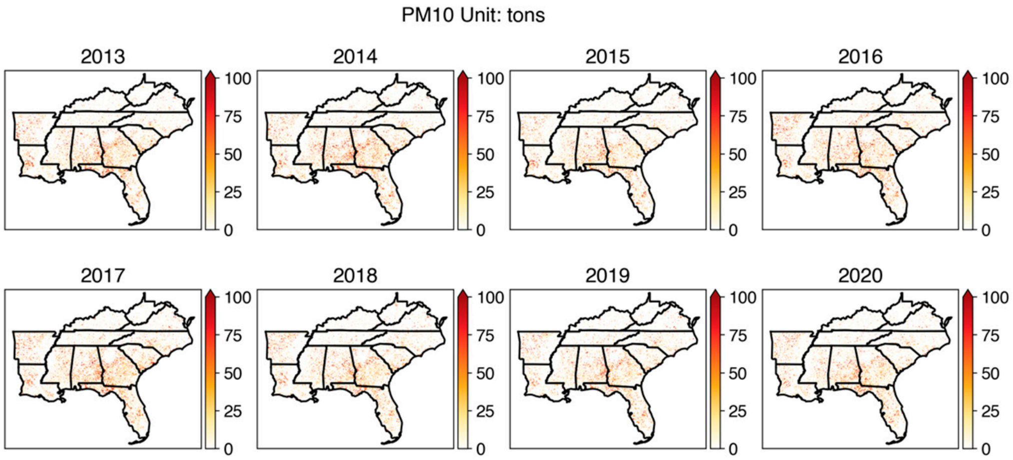

Figure S15. Yearly adjusted-FINN total PM_{10} emission in southeastern U.S. during 2013–2020.

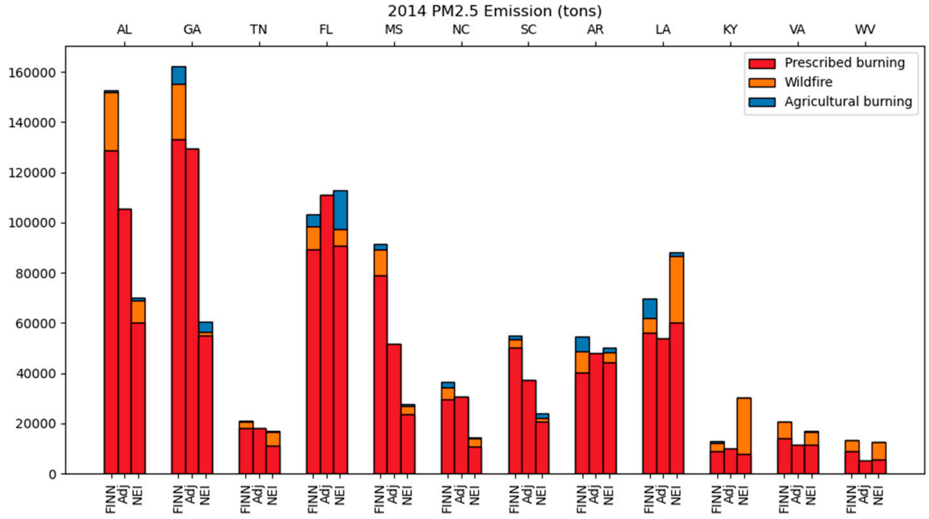

Figure S16. $PM_{2.5}$ Emissions comparisons among FINN, adjusted FINN and NEI for 2014.

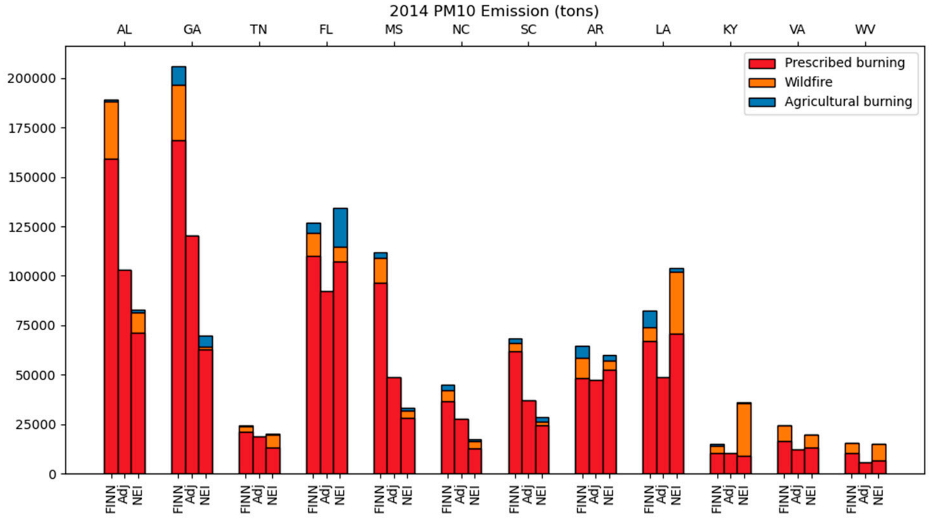

Figure S17. PM_{10} Emissions comparisons among FINN, adjusted FINN and NEI for 2014.

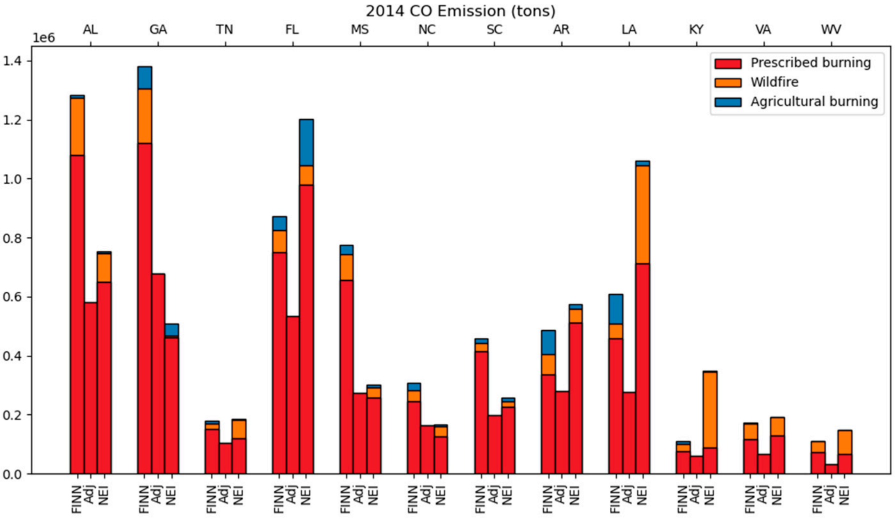

Figure S18. CO Emissions comparisons among FINN, adjusted FINN and NEI for 2014.

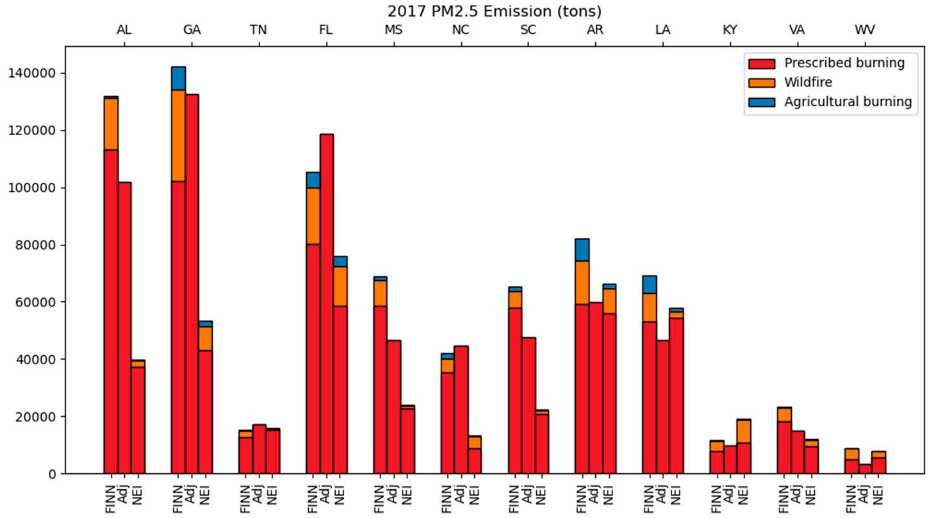

Figure S19. $PM_{2.5}$ Emissions comparisons among FINN, adjusted FINN and NEI for 2017.

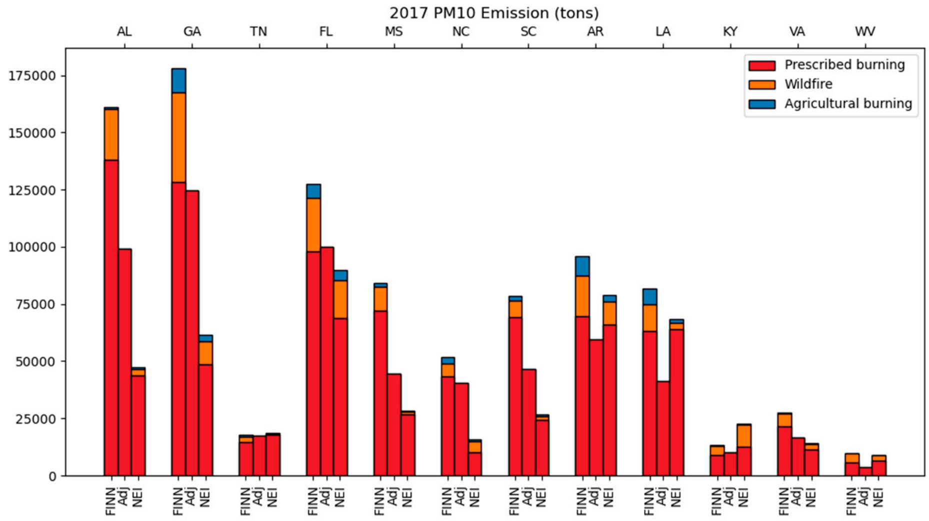

Figure S20. PM_{10} Emissions comparisons among FINN, adjusted FINN and NEI for 2017.

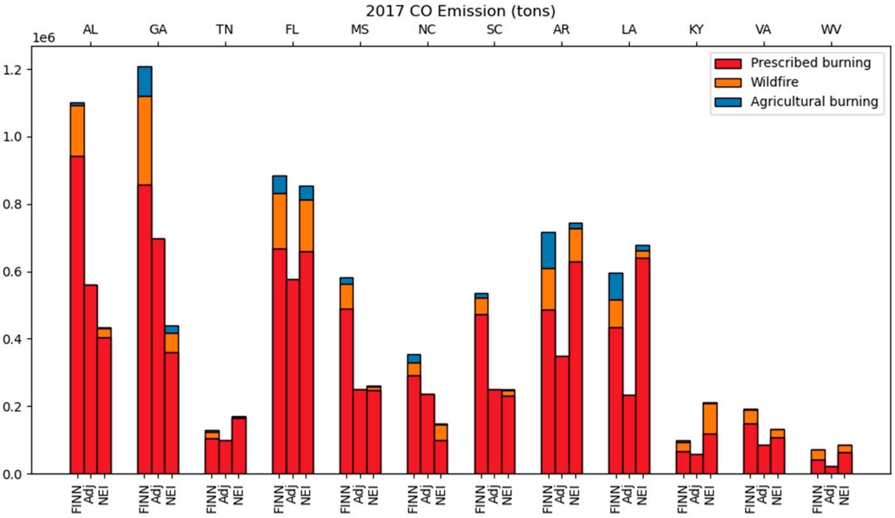

Figure S21. CO Emissions comparisons among FINN, adjusted FINN and NEI for 2017.

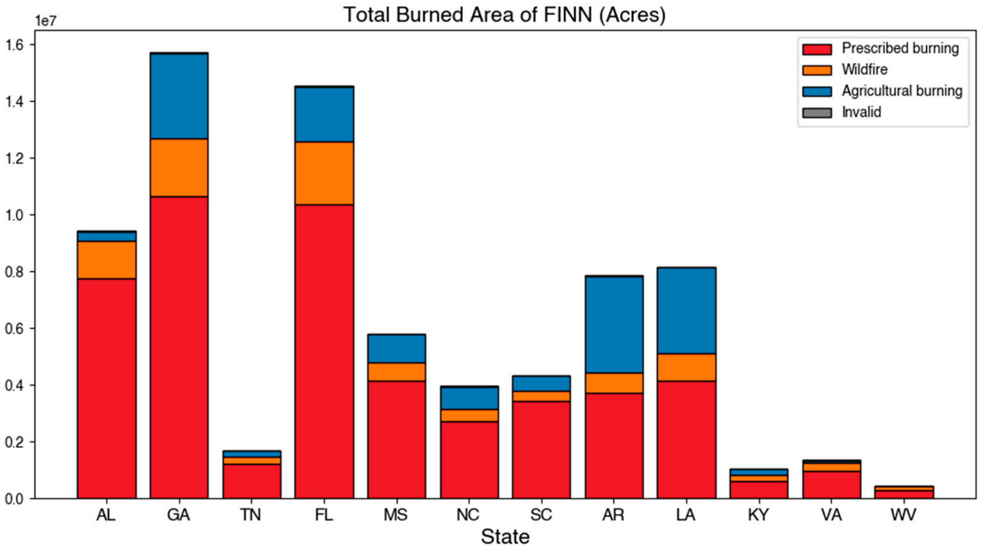

Figure S22. Breakdown of total burned area from FINN by different burn types in southeastern states, 2013–2020.

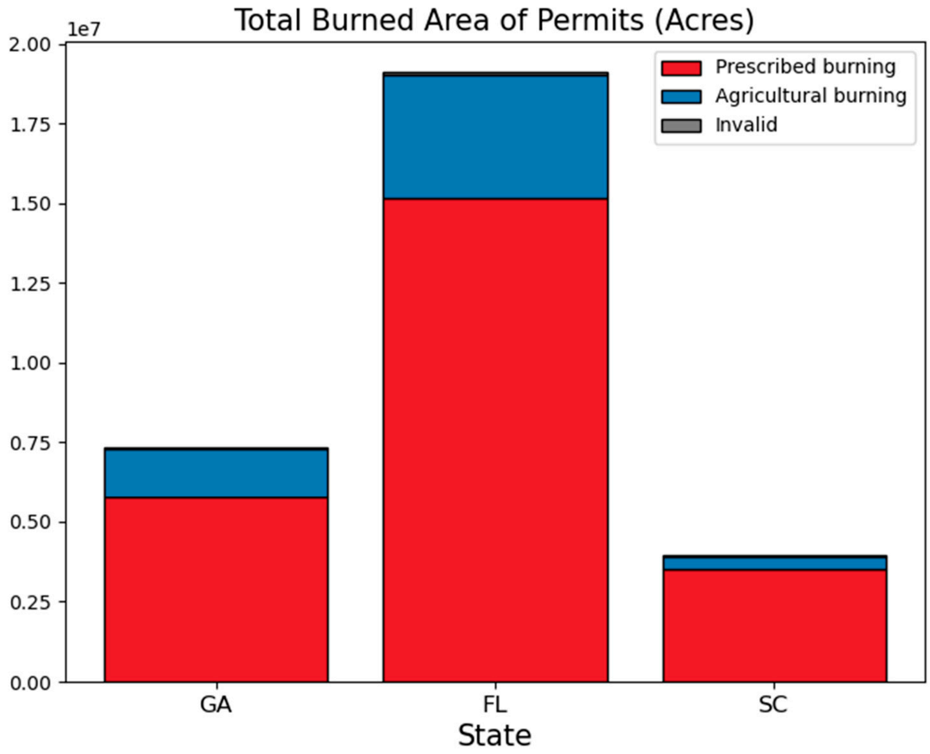

Figure S23. Breakdown of total burned area from permits by different burn types in Georgia, Florida, and South Carolina. Georgia permits cover 2015–2020. Florida and South Carolina permits cover 2013–2020.

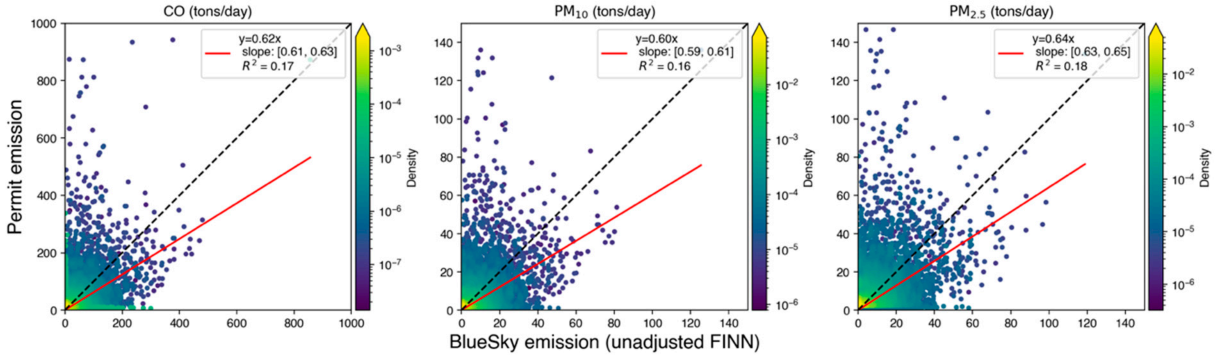

Figure S24. Comparison between permit and BlueSky prescribed burning emissions with unadjusted FINN burned area in matched grid cells under a 4-km grid definition. The black line is a 1:1 line, and the red line is the regression line. Uncertainty of linear regression parameters was reported with a 95% confidence interval.

Study Domain

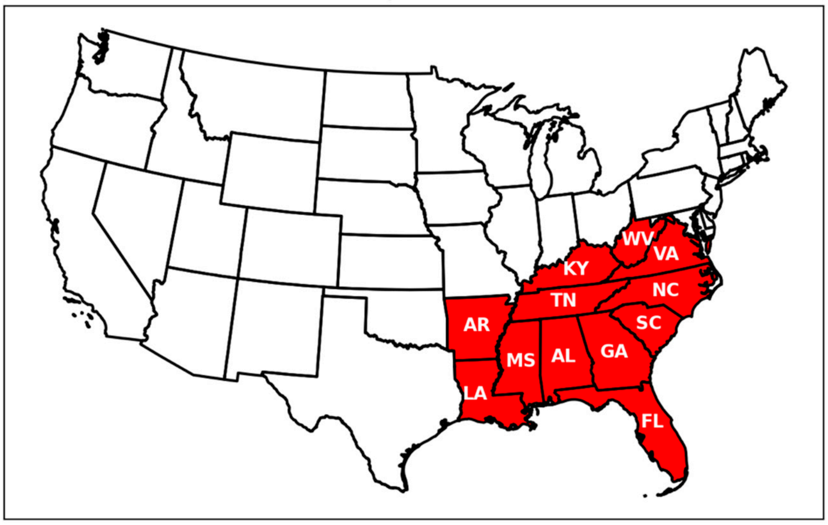

Figure S25. States in the southeastern United States included in the study (State Abbreviations: AL: Alabama; AR: Arkansas; GA: Georgia; FL: Florida; KY: Kentucky; LA: Louisiana; MS: Mississippi; NC: North Carolina; SC: South Carolina; TN: Tennessee; VA: Virginia; WV: West Virginia.)
